# Lighting up the Electrochemiluminescence of Carbon Dots through Pre‐ and Post‐Synthetic Design

**DOI:** 10.1002/advs.202100125

**Published:** 2021-05-11

**Authors:** Francesca Arcudi, Luka Ðorđević, Sara Rebeccani, Michele Cacioppo, Alessandra Zanut, Giovanni Valenti, Francesco Paolucci, Maurizio Prato

**Affiliations:** ^1^ Department of Chemical and Pharmaceutical Sciences INSTM UdR Trieste University of Trieste Via Licio Giorgieri 1 Trieste 34127 Italy; ^2^ Department of Chemistry “Giacomo Ciamician” University of Bologna Via Selmi 2 Bologna 40126 Italy; ^3^ Carbon Bionanotechnology Group Center for Cooperative Research in Biomaterials (CIC biomaGUNE) Basque Research and Technology Alliance (BRTA) Paseo de Miramón 182 Donostia‐San Sebastián 20014 Spain; ^4^ Ikerbasque Basque Foundation for Science Bilbao 48013 Spain; ^5^ Present address: Department of Chemistry Northwestern University 2145 Sheridan Road Evanston IL 60208 USA; ^6^ Present address: Tandon School of Engineering New York University Brooklyn NY 11201 USA

**Keywords:** bottom‐up synthesis, carbon dots, electrochemiluminescence, fluorescence, multicomponent synthesis, nanoparticles, post‐functionalization

## Abstract

Carbon dots (CDs), defined by their size of less than 10 nm, are a class of photoluminescent (PL) and electrochemiluminescent (ECL) nanomaterials that include a variety of carbon‐based nanoparticles. However, the control of their properties, especially ECL, remains elusive and afflicted by a series of problems. Here, the authors report CDs that display ECL in water via coreactant ECL, which is the dominant mechanism in biosensing applications. They take advantage of a multicomponent bottom‐up approach for preparing and studying the luminescence properties of CDs doped with a dye acting as PL and ECL probe. The dependence of luminescence properties on the surface chemistry is further reported, by investigating the PL and ECL response of CDs with surfaces rich in primary, methylated, or propylated amino groups. While precursors that contribute to the core characterize the PL emission, the surface states influence the efficiency of the excitation‐dependent PL emission. The ECL emission is influenced by surface states from the organic shell, but states of the core strongly interact with the surface, influencing the ECL efficiency. These findings offer a framework of pre‐ and post‐synthetic design strategies to improve ECL emission properties, opening new opportunities for exploring biosensing applications of CDs.

## Graphical abstract

Pre‐ and post‐synthetic strategies enhance the electrochemiluminescence (ECL) of carbon dots (CDs). By using a fluorophore and ECL emitter in the bottom‐up synthesis, the major contribution of the surface emitting states to the ECL emission and the role from the states of the core are revealed. These findings have implications for advancing the ECL applications of CDs.

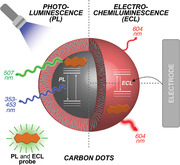

## 1 Introduction

Carbon dots (CDs) are quasi‐spherical nanoparticles with small sizes (<10 nm) composed of carbon, hydrogen, and oxygen atoms, which have received considerable attention as emitting materials.^[^ ^1^, ^2^, ^3^, ^4^ ^]^ The favorable photophysical and electrochemical properties of CDs have led to their emergence as photoluminescent and electrochemiluminescent nanoparticles, with a wide range of applications.^[^ ^5^, ^6^, ^7^, ^8^, ^9^, ^10^ ^]^ Still, they are not a class of materials that can be narrowly defined by their photophysical and electrochemical properties because of the variety of synthetic conditions and precursors. However, the field has made tremendous progress in defining the origin of the photoluminescence (PL) in CDs.^[^ ^11^, ^12^, ^13^, ^14^, ^15^, ^16^ ^]^ The so‐called excitation‐dependent emission is usually emphasized as a common and characteristic feature of amorphous CDs prepared through bottom‐up synthesis. This optical heterogeneity can be explained in terms of i) individual contributions from core and surface functional groups or ii) surface states with the PL activity originating from multiple individual surface‐exposed emitters or *π*–*π* conjugated fragments/molecular fluorophores embedded in the carbon core.^[^ ^17^, ^18^, ^19^, ^20^ ^]^

Compared to the CD photoluminescence investigations, their electrochemiluminescence (ECL) properties have been substantially less explored.^[^ ^5^, ^21^, ^22^ ^]^ On a fundamental level, ECL is a useful characterization technique because it correlates electrochemical and photophysical processes.^[^ ^23^, ^24^ ^]^ On a practical level, the combination of electrochemical and spectroscopic methods leads to a unique sensitivity and signal‐to‐noise ratio, making ECL a leading transduction technique, especially for immunoassays.^[^ ^25^, ^26^, ^27^ ^]^ ECL‐based biosensors represent one of the most promising fields of application of nanomaterials, which can be used either as luminophores or as coreactants.^[^ ^21^, ^28^, ^29^ ^]^ As CDs show ECL activity, the application of CDs in ECL‐based biosensors has progressed faster than fundamental studies on how the CD structures influence the ECL properties. Previous studies reported the ECL properties of carbon‐based dots (amorphous or graphitic and produced by either top‐down or bottom‐up approaches) by both annihilation and coreactant routes.^[^ ^30^, ^31^, ^32^, ^33^, ^34^, ^35^, ^36^ ^]^ Known strategies for modulating the PL have been shown to affect the ECL properties as well.^[^ ^30^, ^31^, ^32^, ^33^, ^34^, ^35^, ^36^ ^]^ For example, the ECL performance of CDs was promoted by introducing surface emissive sites through nitrogen and sulfur doping,^[^ ^35^ ^]^ or reducing non‐radiative recombination and enhancing radiative recombination through the variation of the nitrogen doping concentration.^[^ ^36^ ^]^ Apart from the heteroatom doping strategy, introducing carbon‐related dangling bonds on CDs and modulating the oxidation degree of the CD surface were shown to alter the CD ECL efficiency.^[^ ^30^, ^32^, ^33^, ^34^ ^]^ These studies reported ECL peaks either close or red‐shifted, relative to the PL peak,^[^ ^5^, ^30^, ^31^, ^32^, ^37^ ^]^ but the synthetic/structural rationale behind these different properties is mostly unexplored. Using the same model reported for inorganic nanocrystals, it has been proposed that, while the PL is dominated by excitation and emission within the core, the electron transfer occurs at the surface of CDs, so that the ECL emission is characteristic of the surface energy levels.^[^ ^38^, ^39^, ^40^ ^]^ The variety of synthetic precursors and procedures can affect the CDs’ PL and ECL properties. This has slowed down the understanding of their ECL properties and developing of general models, which could ultimately drive the preparation of nanomaterials with better performance.

**Figure 1:**
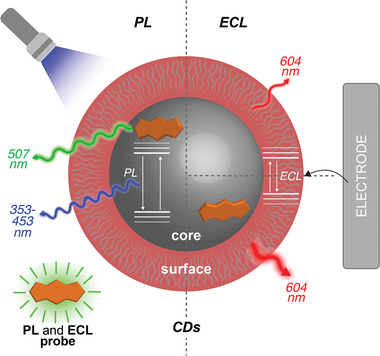
Schematic representation of the ECL and PL mechanism discussed in this work. While the core influences the PL emission, the ECL emission is influenced by the surface states with the core states contributing to the ECL efficiency.

Herein, we report the modulation and improvement of CDs' emission response by precursor design and post‐synthetic modifications. To prepare dye‐doped CDs, we take advantage of a probe, which is capable of acting both as a fluorophore and as ECL emitter. We couple spectroscopic and electrochemical studies to elucidate the contribution to the structure from the precursors and gather information about their core and surface. While the precursors' choice can be used to impart certain core/surface functionalities, this strategy does not achieve control of the surface as effectively as post‐synthetic modification procedures. We, therefore, report post‐synthetic modifications of surface functional groups to probe the CD luminescence properties further. CDs serve as ECL luminophore in water via the coreactant ECL mechanism with efficiencies sensitive to both pre‐ and post‐synthetic approaches. Our data indicate that contributions from core or surface states mainly characterize PL or ECL emission, respectively, though the states of the core strongly interact with the surface affecting the ECL efficiency and the surface‐emitting states from the organic shell influence the efficiency of the blue excitation‐dependent PL emission (**Figure** **1**). Ultimately, this work aims at increasing our understanding on how to master the surface and core properties for the preparation of CDs with improved ECL response in water and open the path to access a library of nanoparticles for biosensing applications.

## 2 Results and Discussion

### 2.1 Pre‐ and Post‐Synthetic Design of (*o*B‐ or *p*B‐)NCDs with Primary or Alkylated Amines

Carbon dots can be prepared through several procedures, which can be categorized into top‐down and bottom‐up routes.^[^ ^4^, ^8^ ^]^ Bottom‐up hydrothermal syntheses usually lead to nanoparticles that possess a carbogenic core (consisting of an amorphous sp^3^ carbon matrix and clusters of sp^2^ carbons) and a surface with various nitrogen‐ and oxygen‐rich functional groups. Several studies, including ours, have proven an organic core/shell type of structure for carbon dots prepared by a bottom‐up approach.^[^ ^2^, ^5^, ^13^, ^41^, ^42^, ^43^, ^44^, ^45^, ^46^, ^47^ ^]^ Here, we build on our approach of using the arginine (Arg) and ethylenediamine (EDA) precursor mixture to produce nitrogen‐doped carbon nanodots (NCDs from now on), which are characterized by an amorphous carbogenic core and a surface rich in primary amino groups.

**Figure 2:**
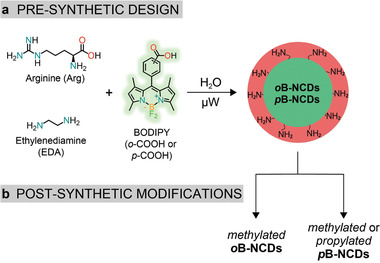
Precursors used for the a) synthesis of *o*B‐NCDs and *p*B‐NCDs and b) post‐synthetic modification of the surface amino groups on the surface.

In this study, we compare the PL and ECL emission of NCDs to that of nanoparticles synthesized from a precursor mixture in which we add a boron‐dipyrromethene (BODIPY) dye as PL and ECL active probe (Figures 1 and **2**). BODIPYs have distinctive absorption/PL emission profiles and have been reported as efficient ECL fluorophores.^[^ ^48^, ^49^, ^50^, ^51^, ^52^ ^]^ Furthermore, we reasoned that a small aromatic precursor (such as the boron‐dipyrromethene unit) would be integrated into the dots under hydrothermal conditions, which has been successfully done with other dyes such naphthalene diimides and porphyrins.^[^ ^53^, ^54^ ^]^ Besides, the structural properties of BODIPYs play an important role in ECL emission.^[^ ^55^, ^56^, ^57^, ^58^, ^59^ ^]^ The BODIPY dyes used in the present work, for example, have been modeled to bear an analogous substitution pattern to those used by Bard and coworkers as ECL luminophores in water.^[^ ^60^ ^]^ To facilitate the incorporation of the dye, we introduced a carboxylate function in a *meso*‐phenyl substituent to promote the formation of amide bonds with the amines from the Arg and EDA mixture under hydrothermal conditions.^[^ ^61^, ^62^ ^]^ Furthermore, the carboxylic group was introduced either at the *ortho* or at the *para* position of the phenyl ring of the BODIPY (hereafter abbreviated as BODIPY‐*o*‐COOH and BODIPY‐*p*‐COOH, Figure 2) to test whether the PL quantum yield of the dye in the nanodots would be affected by this isomerism as it is at the molecular level.^[^ ^48^ ^]^

We have previously shown that NCDs' surface functional groups influence the ECL emission when the carbon dots are used as coreactants.^[^ ^44^ ^]^ For this work, we hypothesize an influence of the surface amines also when the CDs are used as emitters. We analyzed this possibility by comparing the effect on the PL and, especially, ECL emission of the primary amines to that of alkylated amines (Figure 2).

### 2.2 Synthesis and Structural Characterization of (*o*B‐ or *p*B‐)NCDs with Primary or Alkylated Amines

We synthesized small and water‐soluble NCDs using our efficient microwave (MW)‐assisted hydrothermal approach from the Arg and EDA mixture.^[^ ^41^ ^]^ The synthesis of the BODIPY probes that we target is straightforward since the boron‐dipyrromethene core can be obtained in one‐pot synthesis, as detailed in the synthetic procedure part of the Supporting Information. We then prepared *o*B‐NCDs or *p*B‐NCDs by introducing either BODIPY‐*o*‐COOH or BODIPY‐*p*‐COOH in the reaction mixture, optimizing the MW parameters and the molar ratio among the three organic precursors. With appropriate viscosity and temperature controls, it is possible to obtain uniform carbonization through condensation, polymerization, and aromatization processes (see Supporting Information for details).

**Figure 3:**
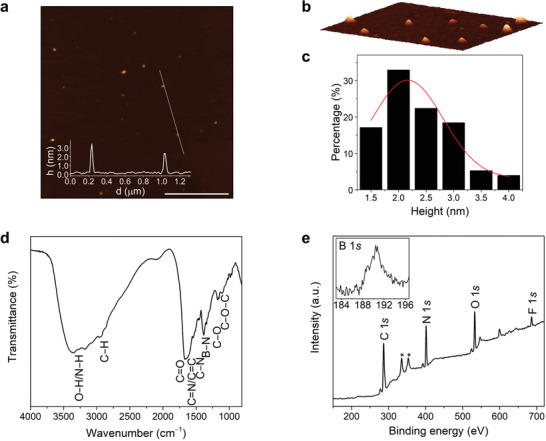
Morphological and structural characterization of *p*B‐NCDs. a) Tapping mode AFM (3.0 × 3.0 µm) from a drop‐cast aqueous solution on a mica substrate (scale bar, 1.0 µm), inset is the height profile along the dashed line; b) 3D close‐up AFM image (0.7 × 0.8 µm); c) size histogram with curve fit using a Gaussian model (FWHM: 1.52); d) FT‐IR spectrum; e) XPS survey spectrum showing the C 1s, N 1s, O 1s, B 1s, and F 1s (deconvoluted spectra are in Figure S2, Supporting Information, *from Au substrate).

Atomic force microscopy (AFM) characterization of *p*B‐NCDs (**Figure** **3**a–c) showed nanoparticles with a round shape and a size of 2.17 ± 0.65 nm (that is consistent with TEM images, see Figure S1, Supporting Information) comparable to NCDs.^[^ ^41^ ^]^ The structural characterization by Fourier‐transform infrared spectroscopy (FT‐IR) (Figure 3d) and X‐ray photoelectron spectroscopy (XPS) (Figure S2, Supporting Information) revealed the presence of many functional groups on their surface, similar to NCDs.^[^ ^41^ ^]^ Interestingly, the peak at 1683 cm^−1^ in the FT‐IR spectrum of the BODIPY precursor, attributed to the carboxylic acid moiety, disappears in the *p*B‐NCDs spectrum, thereby suggesting that the carboxylic acid has reacted with the other precursors (Figure S3, Supporting Information). From the full‐scan XPS spectrum of *p*B‐NCDs (Figure 3e) C 1s at 286.3 eV, N 1s at 401.0 eV, O 1s at 532.3 eV, B 1s at 190.3 eV, F 1s at 685.8 eV are detected. The atomic percentages for C, N, O, B, and F are as follows: 61.1, 22.4, 13.2, 1.2, and 2.1, respectively. The binding energy of the F 1s is consistent with the presence of inorganic fluoride belonging to the BF_2_ group from the boron‐dipyrromethene core,^[^ ^63^, ^64^ ^]^ indicating that this group is stable under our microwave hydrothermal conditions. We confirmed through AFM analysis and FT‐IR spectroscopy of *o*B‐NCDs (Figures S4 and S5, Supporting Information) that the functionalization of the phenyl ring on the BODIPY does not influence the morphology and structural features of the nanoparticles.

The surfaces of (B‐)NCDs are rich in primary amino groups, determined by Kaiser test (synthetic procedures, Supporting Information). These primary amino groups were further converted into methylated and propylated amines through reductive alkylation reactions (Figure 2), either using Eschweiler−Clarke methylation or reductive propylation (propionaldehyde and sodium cyanoborohydride),^[^ ^42^ ^]^ with the reactions monitored using the Kaiser test (synthetic procedures, Supporting Information).

### 2.3 Ground State Absorption and Photoluminescence of (*o*B‐ or *p*B‐)NCDs with Primary or Alkylated amines

**Figure 4:**
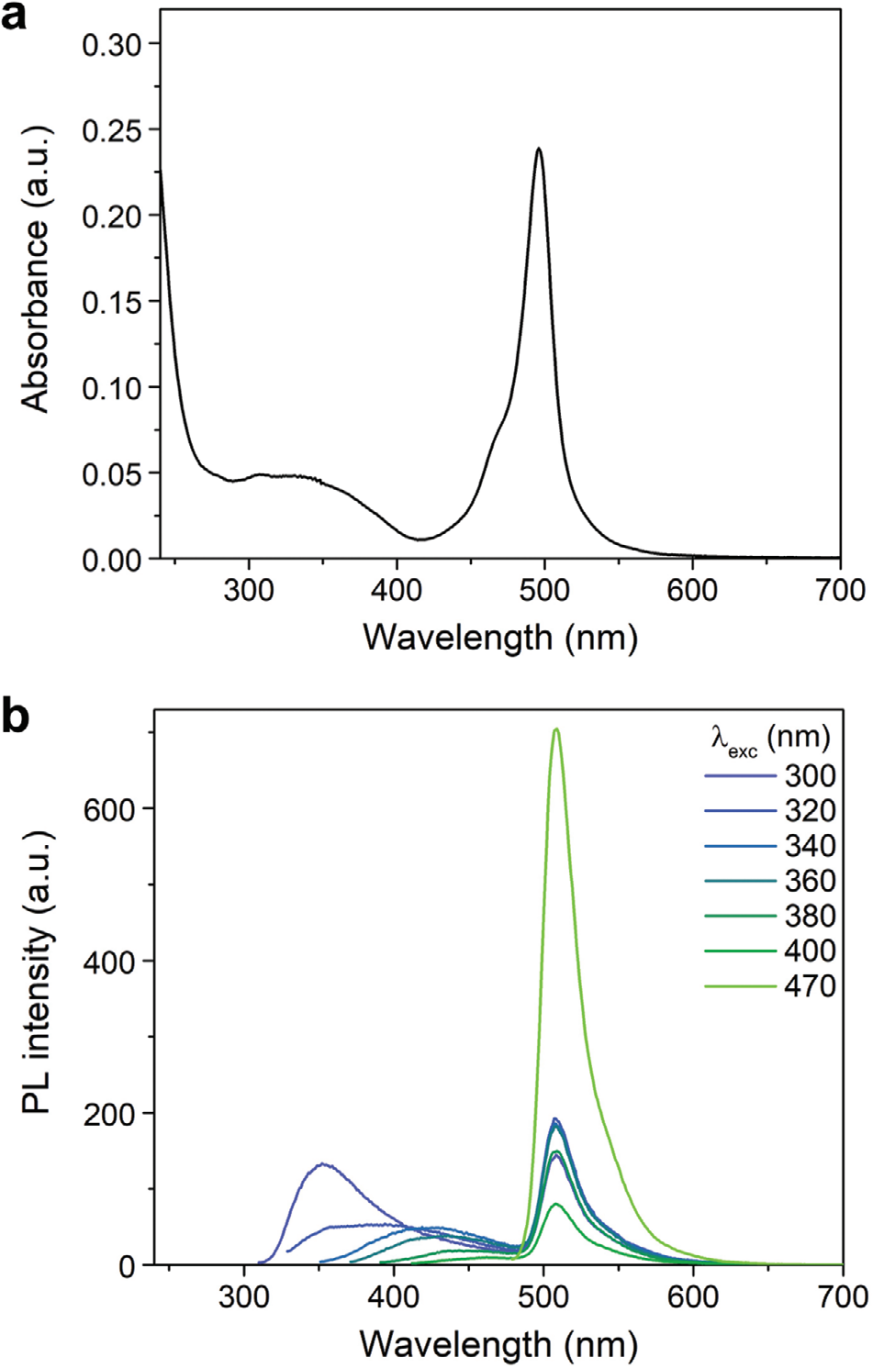
Absorption and photoluminescence spectra of *p*B‐NCDs. Experiments performed in water at 298 K. a) UV–Vis spectrum (5 × 10^−2^ mg mL^−1^) and b) PL emission spectra at different excitation wavelengths.

UV–Vis and PL spectra of B‐NCDs in water provide evidence of the incorporation of the dye in the nanoparticle. The absorption spectrum of *p*B‐NCDs (**Figure** **4**a) is characterized by a strong S_0_→S_1_ (*π*–π*) transition at 496 nm and a weaker broad band around 350 nm that can be attributed to the S_0_→S_2_ transition of the BODIPY moiety and the *π*–π* transition of the C = C units observed for NCDs (Figure S6, Supporting Information).^[^ ^41^, ^48^ ^]^ *p*B‐NCDs show a dual PL spectrum emitting from the blue‐green to the green region upon excitation from 300 to 470 nm (Figure 4b; Figures S6 and S7, Supporting Information).

The first peak shows the typical excitation wavelength‐dependence phenomenon of NCDs that shifts from 353 to 453 nm when the excitation wavelength changes from 300 to 400 nm.^[^ ^41^ ^]^ The second peak centered at 507 nm is assigned to the BODIPY moiety,^[^ ^48^, ^49^, ^51^ ^]^ and the fluorophore protection by the carbonaceous matrix (vide infra) also explains the photostability of the emission from the BODIPY moiety of *p*B‐NCDs (Figure S8, Supporting Information).

We did not observe significant differences in the UV–Vis and PL spectra of *o*B‐NCDs compared to *p*B‐NCDs (Figure S9, Supporting Information). The absorption and emission peak position of the BODIPY moiety is unchanged, which is typical of the boron‐dipyrromethene core, while the emission in the blue region is slightly shifted (by ≈10 nm) than that of *o*B‐NCDs. However, we observed changes in the photoluminescence quantum yield (PLQY) of *o*B‐NCDs compared to *p*B‐NCDs. We calculated the PLQY of the blue emission as 6.0 ± 0.4% and 4.1 ± 0.1% for *p*B‐ and *o*B‐NCDs, respectively. On the other hand, we calculated the PLQY of the green emission as 26.7 ± 0.6% and 43.7 ± 1.2% for *p*B‐ and *o*B‐NCD, respectively, which indicates the same dependence of the green PLQY on the substitution of the phenyl ring observed for the dyes BODIPY‐*o*‐COOH (82.3 ± 3.2%) and BODIPY‐*p*‐COOH (43.3 ± 1.2%) alone (in water; Figure S10, Supporting Information). The absorption and emission features do not considerably change following the modification of the amine surface groups to methylated or propylated amines (Figures S11–S13, Supporting Information). However, we noticed that surface modification reactions affect the blue PLQY to a greater degree than the green PLQY (see below), which is in agreement with analogous observations for NCDs.^[^ ^42^, ^44^ ^]^ We believe that primary, methylated and propylated amines introduce different surface states as surface functional groups and defects,^[^ ^3^, ^5^, ^10^, ^11^ ^]^ which have an effect on the excitation‐dependent blue PL efficiency.

### 2.4 Electrochemistry and Electrochemiluminescence of (*p*B‐)NCDs with Primary Amines

We investigated the electrochemical properties of NCDs and *p*B‐NCDs through cyclic voltammetry (CV) in *N*,*N*‐dimethylformamide (DMF) containing 0.1 м Bu_4_NPF_6_ as supporting electrolyte. The majority of BODIPY dyes show reversible reduction and oxidation waves with peaks near −1.0 and +1.3 V. However, the electrochemical behavior is affected by the BODIPY ligands and can be easily modified by using different moieties on the dye.^[^ ^50^, ^65^ ^]^ BODIPY‐*p*‐COOH showed an irreversible oxidation and a reversible reduction within the potential windows at a potential of +1.14 V (peak potential vs SCE) and −1.09 V (E_1/2_ vs SCE), respectively (Figure S14, Supporting Information). On the other hand, NCDs showed an irreversible oxidation with a peak potential of +1.1 V (vs SCE) ascribed to the amino groups present on the surface (Figure S15, Supporting Information),^[^ ^44^ ^]^ which could be modulated by employing different precursors.^[^ ^66^ ^]^

**Figure 5:**
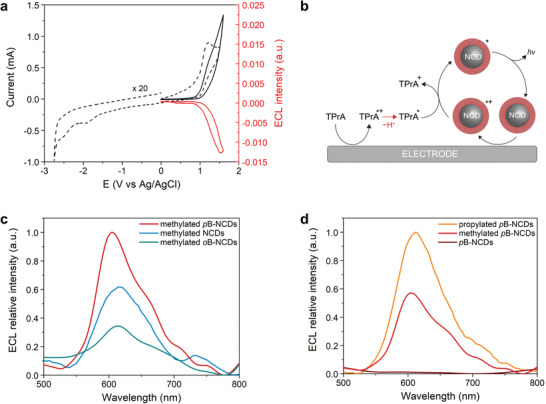
CV and ECL spectra of NCDs and B‐NCDs. a) CV of *p*B‐NCDs in DMF (dashed black line) and CV of *p*B‐NCDs in phosphate buffer 0.2 м and TPA 180 × 10^−3^ m (solid black line) compared to CV‐ECL emission of the same sample (red line). b) ECL emission “oxidative‐reductive” coreactant mechanism hypothesis: both oxidations of TPrA coreactant and (B‐)NCDs emitter occur on the electrode generating the (B‐)NCDs excited states. c) ECL emission of methylated *p*B‐NCDs (1.0 mg mL^−1^, red line), methylated NCDs (1.0 mg mL^−1^, blue line) and methylated *o*B‐NCDs (1.0 mg mL^−1^, green line); d) ECL emission of propylated *p*B‐NCDs (1.0 mg mL^−1^, orange line), methylated *p*B‐NCDs (1.0 mg mL^−1^, red line) and *p*B‐NCDs (1.0 mg mL^−1^, wine line). For (c,d): Experiments performed with TPrA (180 × 10^−3^ m) as coreactant in phosphate buffer (0.2 м) as supporting electrolyte. GC electrode potential referred to Ag/AgCl at room temperature. Platinum wire as counter electrode.

In line with those results, the CV of *p*B‐NCDs displays a well‐defined anodic peak at +1.20 V (vs SCE) (**Figure** **5**a), attributed to the oxidation of the amino groups, with the low anodic current of the BODIPY oxidation being overwhelmed by the signal associated to the amino groups. On the other hand, the availability of BODIPY moieties in *p*B‐NCDs for its direct oxidation/reduction was verified in the cathodic potential windows. In fact, the CV of *p*B‐NCDs showed a new irreversible reduction peak at −1.90 V (vs SCE). From the comparison with NCDs and BODIPY‐*p*‐COOH electrochemical properties, the peak at −1.90 V can be attributed to the BODIPY reduction, while the differences observed in both its potential value (BODIPY reduction occurs at −1.09 V vs SCE) and its kinetics (*p*B‐NCDs show sluggish kinetics) would suggest a considerably different novel nano‐environment surrounding the electroactive dye. The comparison between the redox potentials of *p*B‐NCDs (*E*_ox_ = +1.2 V; *E*_red_ = −1.9 V vs SCE) and NCDs (*E*_ox_ = +1.1 V; *E*_red_ = −2.5 V vs SCE) shows that the presence of the BODIPY moiety reduces the bandgap of *p*B‐NCDs (Δ*E* = 3.1 V) with respect to NCDs (Δ*E* = 3.6 V) thus explaining the observed optical bathochromic shift.

The relatively easy accessibility to the first oxidation, together with the solubility and photoluminescence in water, make B‐NCDs good candidates for ECL in aqueous solutions. Therefore, we exploited the coreactant ECL strategy, which has the advantage of enabling the formation of radical species within the water potential window. The ECL measurments were performed in phosphate buffer (0.2 м, pH = 6.9) using tri‐*n*‐propylamine (TPrA, 180 × 10^−3^ m) as sacrificial coreactant, which follows an “oxidative‐reduction” mechanism.^[^ ^26^, ^67^, ^68^, ^69^ ^]^ We used a constant concentration of 1.0 mg mL^−1^ for the array of B‐NCDs investigated to compare the ECL signal intensities. We also tested the B‐NCDs without the TPrA as coreactant, and we did not observe any ECL signal (see Figure S16, Supporting Information). We chose a glassy carbon (GC) working electrode due to its known capability to provide excellent ECL performance and low surface electrode modification caused by the high potential applied.^[^ ^70^ ^]^ Despite their utility as ECL coreactants,^[^ ^44^ ^]^ NCDs and B‐NCDs rich in surface primary amines have low efficiency as ECL dyes (Figure S17, Supporting Information). Figure 5a shows the current−potential and ECL−potential profiles (CV‐ECL) of *p*B‐NCDs, demonstrating that, while the CV is dominated by the TPrA oxidation (*E* ^0^ = +0.88 V vs SCE) due to its high concentration, the intensity of the ECL‐potential curve increases in correspondence of the anodic oxidation of TPrA and *p*B‐NCDs. The latter observation is typical for an ECL mechanism where both TPrA and fluorophore are oxidized at the electrode surface, exemplified by the scheme reported in Figure 5b.^[^ ^71^ ^]^ Moreover, we studied the ECL signal stability of *p*B‐NCDs and NCDs, both with either primary or methylated amines on the surface, by maintaining constant the potential at the ECL emission maximum (Figures S18 and S19, Supporting Information). Comparison of these ECL curves shows that all nanoparticles follow a regular signal decay, which is due to a combination of different factors, such as electrode oxidation and side reaction connected with TPrA oxidation.^[^ ^70^, ^72^, ^73^ ^]^

### 2.5 Electrochemiluminescence of (*o*B‐ or *p*B‐)NCDs with Alkylated Amines

**Figure 6:**
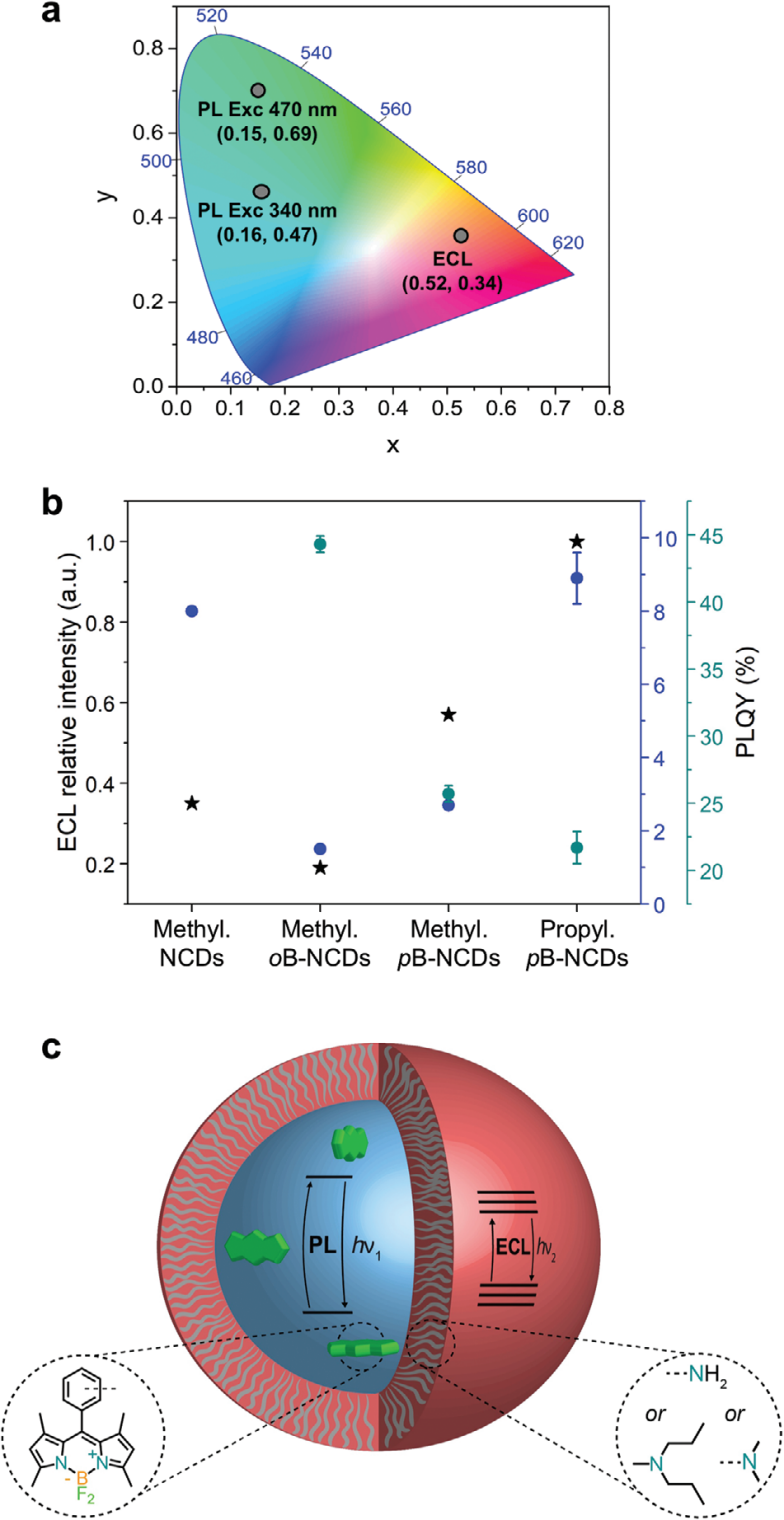
a) 1931 CIE chromaticity diagram showing coordinates from PL (340 nm as excitation wavelength: *x* = 0.16, *y* = 0.47; 470 nm as excitation wavelength: *x* = 0.15, *y* = 0.69) and ECL spectra (*x* = 0.52, *y* = 0.34) of methylated *p*B‐NCDs. b) ECL relative intensity (black stars), blue PLQY (300 nm as excitation wavelength; blue circles) and green PLQY (466 nm as excitation wavelength; green circles) for methylated NCDs, *o*B‐ or *p*B‐NCDs and propylated *p*B‐NCDs. c) Scheme of the core/shell structure of B‐NCDs.

We studied the ECL properties of B‐NCDs modified post‐synthetically to bear either methylated or propylated amines on their surfaces. The surface chemistry might play a vital role in the ECL properties, as it was previously reported in the pioneering work by Bard and coworkers on colloidal quantum dots or silicon nanocrystals.^[^ ^38^, ^44^ ^]^ We did not observe any ECL signal in the absence of TPrA (Figure S16, Supporting Information), as in the case of the B‐NCDs with primary amines. However, when TPrA was used, we found evidence of an intense and stable ECL signal over time for both methylated or propylated NCDs and *o*B‐ or *p*B‐NCDs (Figure S20, Supporting Information), thereby highlighting the importance of surface functional groups in the ECL emission. This observation prompted us to measure the ECL spectra and identify the electrogenerated excited state. The ECL spectrum of methylated *p*B‐NCDs showed a broad peak at ≈604 nm, which is red‐shifted by ≈100 nm from the PL peak ascribed to the BODIPY moiety and by ≈250 nm from the PL peak in the blue region of the spectrum (via excitation at 300 nm) (Figures 5c and 4a; Figure S21, Supporting Information). The extent of red shift is the same as in the ECL spectra of both methylated *o*B‐NCDs and NCDs, which showed ECL peaks at 613 and 615 nm, respectively (Figure 5c). Therefore, we infer that, for post‐synthetically modified dots, the ECL emission is consistent with a charge injection via its surface states (see scheme in **Figure** **6**c). During ECL, both electron and hole need to be separately injected into the dots through its surface; thus, ECL is much more sensitive to the surface states of CDs than the corresponding PL.^[^ ^38^ ^]^ For this reason, specific criteria for water‐soluble design of ECL‐active CDs should be considered to avoid charge carriers from surface states. Figure 5c also compares the ECL intensity for *p*B‐NCDs*, o*B‐NCDs and NCDs, where the surface is rich with methylated amines. The ECL intensity for methylated *p*B‐NCDs is higher than for methylated NCDs by a factor of 1.6. On the other hand, the BODIPY in the methylated *o*B‐NCDs partially quenches the ECL process and the ECL intensity is lower than for methylated *p*B‐NCDs by a factor of 3. A direct comparison of ECL efficiency with the standard [Ru(bpy)_3_]^2+^ and some state‐of‐the‐art ECL dyes (Tables S1 and S2 and Figure S22, Supporting Information) ranks *p*B‐NCDs among the most promising ECL luminophores. Notably, the presence of BODIPY does not affect the ECL wavelength emission. The relative intensities of the ECL spectra (Figure 5c,d) are in agreement with the trend observed in the CV‐ECL (Figures S23 and S24, Supporting Information).

Based on this result, we can conclude that the ECL emission, in terms of the nature of the excited state, depends mainly on the surface properties of carbon dots, though the BODIPY moiety plays a role on the ECL emission efficiency. Furthermore, we investigated the influence of the surface chemistry on the ECL emission based on a comparison of the ECL spectra of *p*B‐NCDs with different amino groups on the surface. Figure 5d shows that the peak wavelength is only slightly affected by the nature of the amino functionalities, although we observe a significant contribution to the ECL efficiency. The ECL intensity for propylated *p*B‐NCDs is higher than for the methylated ones by a factor of 1.7, while *p*B‐NCDs with primary amines do not present any ECL emission signal, as discussed above. We attribute this behavior to the introduction of different surface states by changing the surface functional groups.^[^ ^40^ ^]^ The more efficient emission of the propylated *p*B‐NCDs compared to the methylated ones can be attributed to a better stabilization of the amine‐centered radical cations formed upon oxidation of the emitter.^[^ ^74^ ^]^ The correlation between nanoparticle's structure and ECL activity can also be associated to a better energy alignment between the dye‐doped core and the surface states with a consequent transfer of electronic density on the shell and stabilization of the excited state (Table S3, Supporting Information), making the overall ECL process more efficient. This behavior is consistent with recent results on core/shell and core/shell/shell colloidal quantum dots reported by Su, Peng and coworkers illustrating the importance of designing the band and lattice structure for the ECL emission, which occurs from the surface state and is affected by the core in terms of its efficiency.^[^ ^75^ ^]^ On the other hand, the post‐synthetic modifications have not altered the length of the organic shell considerably, and this rule out the scenario in which surface modifications affect the charge tunneling through the shell.

### 2.6 Comparison of Electrochemiluminescence and Photoluminescence of (*o*B‐ or *p*B‐)NCDs

As complementary techniques to the spectroscopy ones, electrochemical methods have provided evidence on the nature of the emitting state and disentangle the precursor contribution to the emission from the shell and the core. B‐NCDs show a dual blue‐green PL emission, where the excitation‐dependent blue PL emission is characteristic of NCDs, and the green PL emission is due to the BODIPY dye. The ECL spectra of both NCDs and B‐NCDs are substantially red‐shifted with respect to the PL by almost the same amount regardless of the BODIPY dye's presence (Figure 6a). The energetic difference between the PL and the ECL highlights that the emitting states are different. The comparison between PL and ECL of NCDs and B‐NCDs (Figure 6b) provides novel insights for improving the ECL emission of CDs and learning how these choices translate into optical properties changes. The PL and ECL efficiency of NCDs and B‐NCDs (Figure 6b) revealed that i) the surface‐emitting states from the organic shell influence the efficiency of the blue excitation‐dependent PL emission (characteristic of CDs from hydrothermal bottom‐up synthesis), which correlate with the ECL efficiency and ii) the green PL emission is not sensitive to the surface chemistry, but the states of the core, including the nano‐environment of BODIPY, are strictly connected to the organic shell and influence the ECL emission, despite of their primary dependence on surface states from the organic shell. We explain this behavior with a model that features the BODIPY moiety located in the core of the nanoparticle because it contributes to the PL emission (Figure 6c). Still, the comparison of the PLQY and ECL efficiency of NCDs and B‐NCDs with the same surface chemistry revealed that electron and wavefunctions interact strongly with the surface.

## 3 Conclusions

In summary, we reported the design and synthesis of novel dye‐doped CDs demonstrating a framework of precursor design and post‐synthetic modifications to modulate and improve PL and ECL properties. Varying synthetic procedures and precursors would inevitably lead to changes to the CDs’ PL and ECL properties, although principles from this work could still apply. For this reason, we decided to prepare CDs through a multicomponent bottom‐up approach illustrating that different organic precursors contribute to the formation of core and organic shell. We have synthesized and studied NCDs (obtained from Arg and EDA) and B‐NCDs (from Arg, EDA, and BODIPY precursor mixture), with the latter ones doped with a dye capable of serving as both an ECL and PL probe.

We observed ECL from (B‐)NCDs in water in the presence of TPrA as a coreactant. The ECL spectra of all (B‐)NCDs show a peak that is substantially red‐shifted from the PL spectra one and suggest that the emitting states are different. Modifying the amino groups on the surface of (B‐)NCDs, through postfunctionalization reactions, improves the ECL signal, which is evidence of the sensitivity of ECL to CDs surface chemistry. The ECL emission intensity increases from primary amine, to methylated tertiary amines, to propylated tertiary amines. The doping with BODIPY affects the PL properties of CDs, which mainly occur through excitation and emission within the CD core. The ECL maximum wavelength is not sensitive to the presence of the BODIPY but it alters the ECL intensity suggesting that electron and hole wavefunctions can interact strongly with the CD surface.

Our results and model can serve as a tool to guide pre‐ and post‐synthetic approaches to CDs with improved ECL properties, effectively widening their applications. In contrast to other nanoparticles, such as semiconductor nanocrystals, CDs' pre‐synthetic design does not enable fine control of core and surface‐emitting states. Although useful and operationally simple, there are many simultaneous reactions during bottom‐up synthesis, which require high temperature and pressure. Coupling pre‐ and post‐synthetic approaches is crucial for improving the ECL performances. While pre‐synthetic design, targeting *π*‐system conjugation of the core or installing chromophore units, is an option for targeted PL emission, accessing the full toolbox of pre‐ and post‐synthetic approaches would effectively advance their ECL applications. Although CDs are not yet applicable to commercial beads‐based immunoassays, our findings may be generalized to other CDs or inspire novel approaches offering a foundation for rational synthetic strategies to advance ECL applications.

## Conflict of Interest

The authors declare no conflict of interest.

## Supporting information

Supporting Information

## Data Availability

Data available on request from the authors.
