# Supplementary material for: Lighting up the Electrochemiluminescence of Carbon Dots through Pre‐ and Post‐Synthetic Design
Source: Adv Sci (Weinh). 2021 May 11;8(13):2100125. doi: 10.1002/advs.202100125 (PMC8261489; doi:10.1002/advs.202100125)
Supplement: Supplementary file 1 — Supporting Information [file ADVS-8-2100125-s001.pdf]

© 2021 Wiley-VCH GmbH

ADVANCED SCIENCE
Open Access

Supporting Information

for *Adv. Sci.,* DOI: 10.1002/advs.202100125

Lighting up the Electrochemiluminescence of Carbon Dots through Pre- and Post- Synthetic Design

Francesca Arcudi, Luka Đorđević, Sara Rebeccani, Michele Cacioppo, Alessandra Zanut, Giovanni Valenti,* Francesco Paolucci, and Maurizio Prato**

Supporting Information

Lighting up the Electrochemiluminescence of Carbon Dots through Pre- and Post-Synthetic Design

Francesca Arcudi[1,5,†],* Luka Đorđević[1,5,†], Sara Rebeccani[2], Michele Cacioppo[1,3], Alessandra Zanut[2,6], Giovanni Valenti[2],* Francesco Paolucci[2], and Maurizio Prato[1,3,4,*]

[1]Department of Chemical and Pharmaceutical Sciences & INSTM, UdR Trieste, University of Trieste, Via Licio Giorgieri 1, 34127 Trieste, Italy

[2]Department of Chemistry "Giacomo Ciamician", University of Bologna, via Selmi 2, 40126 Bologna, Italy

[3]Carbon Bionanotechnology Group, Center for Cooperative Research in Biomaterials (CIC biomaGUNE), Basque Research and Technology Alliance (BRTA), Paseo de Miramón 182, 20014 Donostia San Sebastián, Spain

[4]Ikerbasque, Basque Foundation for Science, 48013 Bilbao, Spain.

[5]Present address: Department of Chemistry, Northwestern University, 2145 Sheridan Road, Evanston, IL 60208, USA

[6]Present address: Tandon School of Engineering, New York University, Brooklyn, NY 11201, USA.

*Corresponding authors: francescarcudi@gmail.com, g.valenti@unibo.it, prato@units.it

[†]These authors contributed equally to this work.

Table of Contents

1. Materials and Methods 3
2. Synthetic Procedures 6
3. Supplementary Figures and Tables... 12
4. References.. 30

1. Materials and Methods

Chemicals were purchased from Sigma Aldrich, TCI, Acros Organics, Fluorochem and Alfa Aesar and used as received. **Solvents** were purchased from Sigma Aldrich, VWR and Acros Organics, and **deuterated solvents** from Sigma Aldrich, Cambridge Isotope Laboratories and VWR. Generally, dry solvents were purchased and used without further purification. 37% w/v aqueous formalin solution was prepared according to a reference protocol.[1] Kaiser test kit was purchased from Sigma-Aldrich. Dialysis tubes with molecular weight cutoff 0.5-1 kDa were bought from Spectrum Labs. Ultrapure fresh water obtained from a Millipore water purification system (Milli-Q with >18 MΩ × cm resistivity, Millipore) was used in all experiments (unless otherwise specified).

Microwave synthesis (MW) was performed on a CEM Discover-SP microwave reactor.

Thin layer chromatography (TLC) was conducted on pre-coated aluminum sheets with 0.20 mm Merck Silica Gel F254.

Column chromatography was carried out using Merck silica gel 60 Å (particle size 40-63 μm).

Melting points (m.p.) were measured on a Büchi SMP-20 in open capillary tubes and have not been corrected.

NMR spectra were obtained on a *Varian Inova* spectrometer (500 MHz ^{1}H, 126 MHz ^{13}C, 376 MHz ^{19}F) at room temperature (298 K). Chemical shifts are reported in ppm using the solvent residual signal as an internal reference (Chloroform-*d*: $\delta_H = 7.26$ ppm, $\delta_C = 77.23$ ppm; Dimethylsulfoxide-*d*$_6$: $\delta_H = 2.50$ ppm, $\delta_C = 39.51$ ppm; Acetone-*d*$_6$: $\delta_H = 2.05$ ppm, $\delta_C = 29.92$ ppm). The resonance multiplicity is described as *s* (singlet), *d* (doublet), *t* (triplet), ..., *m* (multiplet), *bs* (broad singlet).

Mass spectrometry. Electrospray Ionization (ESI, 5600 eV) mass analysis was performed on a Perkin-Elmer API1.

IR spectra (KBr) were recorded on a Perkin Elmer 2000 spectrometer.

Atomic force microscopy. AFM images were recorded on a Nanoscope IIIa VEECO Instruments microscope. AFM analyses were performed using a HQ:NSC19/ALBS probe (80kHz; 0.6 N m^{-1}) (MikroMasch) from drop cast of samples in an aqueous solution (concentration of few mg mL^{-1}) on a mica substrate. The AFM-images were examined using Gwyddion 2.50 as AFM-image analysis software.

Transmission electron microscopy. TEM images were obtained on a JEOL JEM-1400PLUS transmission electron microscope operating at an acceleration voltage of 120 kV. TEM analyses were performed from drop cast of samples in an aqueous solution (concentration of few mg mL^{-1}) on a carbon-coated 300 mesh copper grid. The TEM-images were examined using Gatan Microscopy Suite as TEM-image analysis software.

Photophysical investigations. Absorption spectra of compounds were recorded on air-equilibrated solutions at room temperature with an Agilent Cary 5000 UV-Vis spectrophotometer, using quartz cells with path length of 1.0 cm. Emission spectra were recorded on an Agilent Cary Eclipse fluorescence spectrofluorometer.

Photoluminescence Quantum Yield. The quantum yield (PLQY) measurements were performed using the relative determination, which is based on the comparison of the integral emission spectra of the sample and the standard (obtained under identical measurement conditions) for solutions of known absorbances or absorption factors at the excitation wavelength.[2] All samples were measured in water. We used Quinine Sulfate (QS, Φ = 0.54 in 0.1 M aq. H_2SO_4) or Fluorescein (F, Φ = 0.89 in 0.1 M aq. NaOH) as the standards for the QY of the blue and the green emission, respectively.[2] We selected 300 nm and 466 nm as excitation wavelength for the QY of the blue and the green emission, respectively, according to published protocols and standards.[2] The fluorescence quantum yields were then calculated according to the next equation:

$$\Phi_x = \Phi_{st} \cdot \frac{I_x}{I_{st}} \cdot \frac{f_{st}}{f_x} \cdot \frac{\eta_x^2}{\eta_{st}^2}$$

I is the measured integrated fluorescence emission intensity, f is the absorption factor, η is the refractive index of the solvent and Φ is the quantum yield. The index x denotes the sample, and the index st denotes the standard. Each value of the QY is an average of three independent measurements and calculations.

PL excited state lifetime. PL excited state lifetime were acquired using TCSPC in a miniTau fluorescence lifetime spectrometer (Edinburgh Instruments) equipped with an EPLED 340 pulsed diode, an EPL445 picosecond pulsed diode laser and high speed PMT detector. Samples were prepare using HPLC grade water. Decays were fit with the Fluoracle software of the instrument using reconvolution analysis. The goodness-of-fit was assessed by a minimized chi-square ($\chi^2 \approx 1.000$) and by visual inspection of the weighted residuals.

ECL measurements. ECL measurements were carried out with PGSTAT30 Ecochemie AUTOLAB instrument in three electrodes home-made transparent plexyglass cell using glassy carbon (GC) as working electrode, a Pt wire as counter electrode and Ag/AgCl, KCl (3 M) as reference electrode. GC was polished with 0.3 μm diamond paste (Struer) before each measurement.

The ECL signal generated by performing the potential step programs was measured with a photomultiplier (PMT) tube Acton PMT PD471 placed at a constant distance in front of the cell and inside a dark box. A voltage of 750 V was supplied to the PMT. The light/current/voltage curves were recorded by collecting the preamplified PMT output signal (by an ultralow-noise Acton research model 181) with the second input channel of the ADC module of the AUTOLAB instrument. Current is recorded by the picoamperometer Keithley 6485 with a current amplification of 00.0 μA.

The ECL spectra were performed by spectrometer/detector interface SpectraHub and by monochromator Acton Spectrapro 2300i, applying a constant potential of 1.6 V using PMT at 750 V of potential amplification, scanning from 400 to 800 nm with a step of 3 nm.

ECL relative efficiency of CDs. The ECL relative efficiency (Φ_{ECL} in %) was measured by following a reported method, and $[Ru(bpy)_3]^{2+}$/TPrA were used as a reference.[3]

The relative ECL efficiency (Φ_{ECL}) was calculated using the relation below:

$$\Phi_x = 100 \times \frac{[\int ECL\, dt / \int i\, dt]_x}{[\int ECL\, dt / \int i\, dt]_{st}}$$

where *x* and *st* correspond to the tested CDs and the reference compound $[Ru(bpy)_3]^{2+}$, respectively. Relative ECL efficiency (Φ_{ECL}) are reported in Table S1.

2. Synthetic Procedures

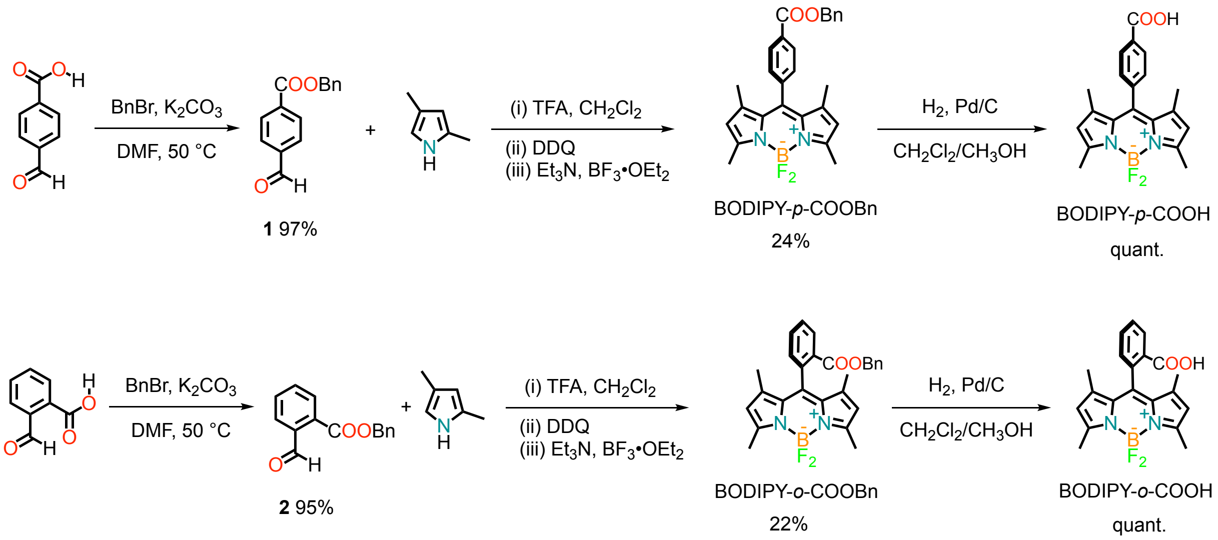

Scheme S1. Synthetic scheme for the preparation of BODIPY-COOH derivatives.

Benzyl 4-formylbenzoate (1)

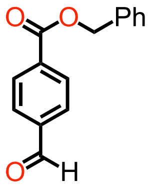

To a solution of 4-formylbenzoic acid (2.20 g, 14.65 mmol) in anhydrous DMF (15.0 mL) was added K_2CO_3 (2.02 g, 14.65 mmol) and the mixture was stirred for 30 min at 50 °C, under argon. Then, benzyl bromide (1.58 mL, 13.32 mmol) was added dropwise, and the mixture was stirred for 3 h at 50 °C under argon. The mixture was diluted with H_2O and Et_2O, the organic phase was washed with sat. aq. K_2CO_3 (2×), brine and H_2O. The organic phase was dried (Na_2SO_4) and the solvent was removed under reduced pressure. The product solidifies on standing and was obtained as white solid (3.09 g, 97% yield). m.p. 37-38 °C. **^{1}H-NMR** (500 MHz, Chloroform-*d*, 25 °C): δ = 10.10 (s, 1H, C*H*O), 8.23 (d, ^3J(H,H) = 8.4 Hz, 2H, Ph-*H*), 7.95 (d, ^3J(H,H) = 8.5 Hz, 2H, Ph-*H*), 7.48 – 7.44 (m, 2H, Ph-*H*), 7.43 – 7.35 (m, 3H, Ph-*H*), 5.40 (s, 2H, CH_2). **^{13}C-NMR** (126 MHz, Chloroform-*d*, 25 °C): δ = 191.8, 165.6, 139.4, 135.7, 135.3, 130.5, 129.7, 128.9, 128.7, 128.6, 67.5. **ES** (MS+): $C_{15}H_{12}O_3$ requires 240.1, found 263.1 $[M+Na]^+$, 295.1 $[M+Na+CH_3OH]^+$. **IR** (KBr): cm^{-1} = 3066, 3036, 2965, 2846, 1720, 1705, 1577, 1503, 1453, 1371, 1272, 1203, 1097, 909, 853, 846, 801, 760, 752, 695. Characterization in accordance with literature.[4]

8-{4-[(benzyloxy)carbonyl]phenyl}-1,3,7,9-tetramethyl-BODIPY (BODIPY-*p*-COOBn)

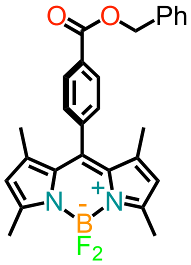

In a 0.5 L round-bottomed flask, a solution of benzyl 4-formylbenzoate (**1**, 2.40 g, 9.99 mmol) and 2,4-dimethylpyrrole (2.06 mL, 19.97 mmol) in CH_2Cl_2 (0.25 L) was prepared and stirred for 20 min at r.t., under argon. Then, trifluoroacetic acid (one drop, catalytic) was added and the mixture was left stirring overnight, under dark and argon. Then, a solution of 2,3-dichloro-5,6-dicyano-1,4-benzoquinone (2.27 g, 10.00 mmol) in CH_2Cl_2 (0.1 L) was added and the mixture was stirred for 1.5 h. Then was added Et_3N (30.0 mL, 215.3 mmol), followed by slow addition of $BF_3{\cdot}OEt_2$ (30.0 mL, 243.1 mmol) and the mixture was left stirring for 2 h. The mixture was diluted with H_2O and the organic phase was washed with H_2O (2×), 1 M aq. HCl solution (5×) and brine. The organic phase was dried (Na_2SO_4) and the solvent was removed under reduced pressure. The mixture was purified by two consecutive column chromatographies (SiO_2, 40-63 µm, Pet. Et./CH_2Cl_2 9:1 → 6:4 v/v) to obtain pure product as bright orange solid (1.10 g, 24% yield).

m.p. 133-134 °C. **^{1}H-NMR** (500 MHz, Chloroform-*d*, 25 °C): δ = 8.21 (d, ^3J(H,H) = 8.3 Hz, 2H, Ph-*H*), 7.52 – 7.48 (m, 2H, Ph-*H*), 7.45 – 7.35 (m, 5H, Ph-*H*), 5.99 (s, 2H, Py-*H*), 5.40 (s, 2H, CH_2), 2.56 (s, 6H, CH_3), 1.35 (s, 6H, CH_3). **^{13}C-NMR** (126 MHz, Chloroform-*d*, 25 °C): δ = 166.0, 156.2, 143.1, 140.4, 140.2, 135.9, 131.1, 131.0, 130.7, 128.9, 128.7, 128.7, 128.6, 121.7, 67.4, 14.8, 14.8. **^{19}F-NMR** (376 MHz, Chloroform-*d*, 25 °C): δ = −146.3 (q, ^1J(B,F) = 31.8 Hz, BF_2). **IR** (KBr): cm^{-1} = 2960, 2925, 2855, 1724, 1542, 1509, 1472, 1405, 1307, 1272, 1194, 1153, 1103, 1085, 1050, 1021, 978, 737, 696. **ES** (MS+): $C_{27}H_{25}BF_2N_2O_2$ requires 458.2, found 439.1 $[M-F]^+$, 497.2 $[M+K]^+$. Characterization in accordance with literature.[5]

4. 8-(4-Carboxyphenyl)-1,3,5,7-tetramethyl-BODIPY (BODIPY-*p*-COOH)

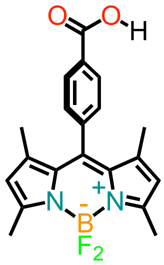

A solution of BODIPY-*p*-COOBn (1.00 g, 2.17 mmol) in CH_2Cl_2/CH_3OH (0.35 L, 1:1 v/v) was degassed with argon for 30 min. Then, Pd/C (0.10 g, 10%) was added under argon flow. The flask was then filled with H_2 (by 3 cycles of evacuating under vacuum and purging with H_2) and the mixture was left stirring at r.t., for 4 h. The mixture was then filtered over a 0.1 μm Teflon membrane, the membrane was then washed with CH_2Cl_2/CH_3OH (20 mL, 1:1 v/v) and the filtrate was concentrated under reduced pressure. Product was obtained as red solid (0.80 g, quantitative yield).
m.p. >250 °C. **^{1}H-NMR** (500 MHz, Dimethylsuldoxide-d_6, 25 °C): δ = 13.28 (bs, 1H, COO*H*), 8.10 (d, ^3J(H,H) = 8.3 Hz, 2H, Ph-*H*), 7.53 (d, J = 8.3 Hz, 2H, Ph-*H*), 6.20 (s, 2H, Py-*H*), 2.46 (s, 6H, CH_3), 1.33 (s, 6H, CH_3). **^{13}C-NMR** (126 MHz, Dimethylsuldoxide-d_6, 25 °C): δ = 166.9, 155.2, 142.6, 140.8, 138.3, 130.3, 130.1, 128.3, 121.6, 14.2, 14.1. **^{19}F-NMR** (376 MHz, Dimethylsuldoxide-d_6, 25 °C): δ = −146.65 (q, ^1J(B,F) = 31.8 Hz). **MS** (ES+): $C_{20}H_{19}BF_2N_2O_2$ requires 368.2, found 349.2 $[M-F]^+$, 391.1 $[M+Na]^+$, 407.1 $[M+K]^+$. **IR** (KBr): cm^{-1} 2975, 2930, 2860, 1683, 1610, 1549, 1542, 1516, 1471, 1436, 1412, 1370, 1314, 1291, 1281, 1260, 1199, 1157, 1124, 1091, 1079, 1048, 1021, 985, 973, 813, 766, 739. Characterization in accordance with literature.[6]

Benzyl 2-formylbenzoate (**2**)

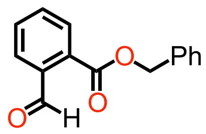

To a solution of 2-formylbenzoic acid (2.00 g, 13.33 mmol) in anhydrous DMF (15.0 mL) was added K_2CO_3 (2.09 g, 15.12 mmol) and the mixture was stirred for 30 min at 50 °C, under argon. Then, benzyl bromide (1.50 mL, 12.62 mmol) was added dropwise and the mixture was stirred for 3 h at 50 °C, under argon. The mixture was diluted with H_2O and Et_2O, the organic phase was washed with sat. aq. $NaHCO_3$ (2×), brine and H_2O. The organic phase was dried (Na_2SO_4) and the solvent was removed under reduced pressure. The product was obtained as a pale-yellow oil (3.04 g, 95% yield).
^{1}H-NMR (500 MHz, Chloroform-*d*, 25 °C): δ = 10.63 (s, 1H, C*H*O), 8.01 – 7.99 (m, 1H, Ph-*H*), 7.95 – 7.93 (m, 1H, Ph-*H*), 7.67 – 7.62 (m, 2H, Ph-*H*), 7.46 – 7.35 (m, 5H, Ph-*H*), 5.42 (s, 2H, CH_2). **^{13}C-NMR** (126 MHz, Chloroform-*d*, 25 °C): δ = 192.0, 166.1, 137.1, 135.3, 132.9, 132.4, 132.0, 130.4, 128.7, 128.6, 128.6, 128.5, 67.7. **ES** (MS+): $C_{15}H_{12}O_3$ requires 240.1, found 263.1 $[M+Na]^+$, 295.1 $[M+Na+CH_3OH]^+$. **IR** (KBr): cm^{-1} = 3067, 3035, 2957, 2891, 1713, 1697, 1596, 1576, 1497, 1453, 1377, 1265, 1194, 1128, 1072, 961, 912, 830, 798, 749, 696, 640.

8-{2-[(benzyloxy)carbonyl]phenyl}-1,3,7,9-tetramethyl-BODIPY (BODIPY-*o*-COOBn)

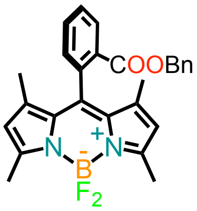

In a 0.5 L round-bottomed flask, a solution of benzyl 2-formylbenzoate (**2**, 1.21 g, 5.04 mmol) and 2,4-dimethylpyrrole (1.04 mL, 10.1 mmol) in CH_2Cl_2 (0.20 L) was prepared and stirred for 20 min at r.t., under argon. Then, trifluoroacetic acid (one drop, catalytic) was added and the mixture was left stirring overnight, under dark and argon. Then, a solution of 2,3-dichloro-5,6-dicyano-1,4-benzoquinone (1.13 g, 5.00 mmol) was added, and the mixture was stirred for 1.5 h. Then was added Et_3N (9.6 mL, 69.3 mmol), followed by slow addition of $BF_3{\cdot}OEt_2$ (10.4 mL, 84.2 mmol) and the mixture was left stirring overnight. To the mixture was added H_2O and the organic phase was washed with H_2O (2×), 0.1 M $NaHCO_3$ (2×) and brine. The organic phase was dried (Na_2SO_4) and the solvent was removed under reduced pressure. The mixture was purified by two consecutive column chromatographies (SiO_2, 40-63 µm, Pet. Et./CH_2Cl_2 9:1 → 3:1 v/v) to obtain pure product as bright orange solid (0.51 g, 22% yield). m.p. 47-49 °C. **^{1}H-NMR** (500 MHz, Chloroform-*d*, 25 °C): δ = 8.11 (dd, $^{3,4}J$(H,H) = 7.9, 1.2 Hz, 1H, Ph-*H*), 7.64 (td, $^{3,4}J$(H,H) = 7.5, 1.4 Hz, 1H, Ph-*H*), 7.56 (td, $^{3,4}J$(H,H) = 7.7, 1.3 Hz, 1H, Ph-*H*), 7.32 (dd, $^{3,4}J$(H,H) = 7.6, 1.3 Hz, 1H, Ph-*H*), 7.25 – 7.24 (m, 3H, Ph-*H*), 7.14 – 7.12 (m, 2H, Ph-*H*), 5.88 (s, 2H, Py-*H*), 5.13 (s, 2H, C*H*$_2$), 2.55 (s, 6H, C*H*$_3$), 1.27 (s, 6H, C*H*$_3$). **^{13}C-NMR** (126 MHz, Chloroform-*d*, 25 °C): δ = 166.0, 155.0, 142.1, 141.1, 135.6, 135.1, 132.9, 131.0, 130.5, 129.7, 129.5, 129.2, 128.5, 128.3, 121.1, 121.0, 67.5, 14.7, 14.0. **ES** (MS+): $C_{27}H_{25}BF_2N_2O_2$ requires 458.2, found 439.1 $[M-F]^+$, 497.2 $[M+K]^+$. **IR** (KBr): cm^{-1} = 2958, 2921, 2851, 1720, 1632, 1547, 1508, 1472, 1409, 1383, 1368, 1308, 1285, 1257, 1195, 1155, 1121, 1084, 1048, 979, 734, 696.

8-(2-Carboxyphenyl)-1,3,5,7-tetramethyl-BODIPY (BODIPY-*o*-COOH)

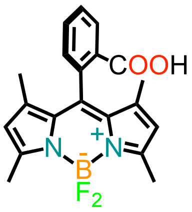

A solution of BODIPY-*o*-COOBn (0.40 g, 0.87 mmol) in CH_2Cl_2/CH_3OH (0.12 L, 1:1 v/v) was degassed with argon for 30 min. Then, Pd/C (0.04 g, 10%) was added, under argon flow. The flask was then filled with H_2 (by 3 cycles of evacuating under vacuum and purging with H_2) and the mixture was left stirring at r.t., for 1 h. The mixture was then filtered over a 0.1 µm Teflon membrane, the membrane was then

washed with CH_2Cl_2/CH_3OH (20 mL, 1:1 v/v) and the filtrate was concentrated under reduced pressure. Product was obtained as red solid (0.32 g, quantitative yield).

m.p. >250 °C. **^{1}H-NMR** (500 MHz, Acetone-d_6, 25 °C): δ = 11.45 (bs, 1H, COO*H*), 8.19 (d, ^3J(H,H) = 7.9 Hz, 1H, Ph-*H*), 7.84 (t, ^3J(H,H) = 7.6 Hz, 1H, Ph-*H*), 7.74 (t, ^3J(H,H) = 7.7 Hz, 1H, Ph-*H*), 7.47 (d, ^3J(H,H) = 7.6 Hz, 1H, Ph-*H*), 6.08 (s, 2H, Py-*H*), 2.50 (s, 6H, CH_3), 1.38 (s, 6H, CH_3). **^{13}C-NMR** (126 MHz, Acetone-d_6, 25 °C): δ = 166.1, 154.4, 142.6, 142.0, 135.8, 133.1, 130.9, 130.8, 130.8, 129.7, 129.3, 120.8, 13.6, 13.3. **MS** (ES+): $C_{20}H_{19}BF_2N_2O_2$ requires 368.2, found 349.2 $[M-F]^+$, 391.1 $[M+Na]^+$, 407.1 $[M+K]^+$. **IR** (KBr): cm^{-1} = 3319, 1727, 1540, 1508, 1469, 1440, 1405, 1370, 1300, 1251, 1226, 1197, 1158, 1135, 1102, 1082, 981, 933, 823, 799, 775, 734, 688, 64. Characterization in accordance with literature.[7]

***p*B-NCDs**

A solution of L-arginine (L-Arg, 40.0 mg, 230 μmol), BODIPY-*p*-COOH (22.0 mg, 59.8 μmol) and ethylenediamine (EDA, 8 μL, 115 μmol) in MilliQ water (70 μL) was heated by microwave at 200 °C, 5 psi, 200 W for 180 seconds. After the heating process, the mixture color changed from yellow to red as result of the formation of CDs. The reaction mixture was then diluted with few milliliters of water and filtered through a 0.1 μm microporous membrane. The clear reddish solution was pH corrected from pH 10.0 to pH 7.2, until the solution becomes opalescent, and filtered once again through a 0.1 μm microporous membrane (the precipitate can be readily dissolved in MeOH and the resulting solution shows the typical absorbance and photoluminescence features of BODIPY). The obtained deep orange filtrate solution was then dialyzed against pure water through a dialysis membrane (molecular weight cutoff 0.5-1KDa) for 48 h. We confirmed the effectiveness of this purification by performing a size-exclusion chromatography (SEC) of the obtained material that gave nanoparticles doped with BODIPY as the only fractions. The purified solution was then lyophilized obtaining a red solid (20.0 mg, Kaiser Test 900 $\mu mol_{NH2}\ g^{-1}$), which is stored in a desiccator until characterization or use.

***o*B-NCDs** were synthetized using the same procedure and stoichiometric ratios used for of *p*B-NCDs. The resulting product was obtained as red solid (21.0 mg; Kaiser Test 650 $\mu mol_{NH2}\ g^{-1}$), which is stored in a desiccator until characterization or use.

Methylated *p*B-NCDs

$NaBH_3CN$ (33 mg) was added to a solution of *p*B-NCDs (18.0 mg), formalin (37% aqueous solution, 1.5 mL) in MeCN (1.5 mL). Then acetic acid (77 μL) was added, and the resulting solution was left stirring at r.t., for 24 h. The resulting mixture was dried under reduced pressure, obtaining a red solid.

This residue was dissolved in MeOH and purified by size exclusion chromatography (Sephadex® LH-20), dried under vacuum and lyophilized from water obtaining a red solid (19.6 mg; Kaiser Test 70 μmol_{NH2} g^{-1}), which is stored in a desiccator until characterization or use.

Methylated *o*B-NCDs

In a similar fashion to methylated *p*B-NCDs synthesis, a solution of $NaBH_3CN$ (22.0 mg), *o*B-NCDs (18.0 mg), formalin (37% aqueous solution, 1.0 mL) and acetic acid (51 µL) in MeCN (1.0 mL) was left stirring at r.t., for 24 h. The resulting mixture was dried under vacuum obtaining a red solid. This residue was dissolved in MeOH and purified by size exclusion chromatography (Sephadex® LH-20), dried under vacuum and lyophilized from water obtaining a red solid (18.9 mg; Kaiser Test 20 μmol_{NH2} g^{-1}), which is stored in a desiccator until characterization or use.

Propylated *p*B-NCDs

Similarly to the methylated *p*B-NCDs synthesis, a solution of $NaBH_3CN$ (33.0 mg), *p*B-NCDs (20.0 mg), propionaldehyde (0.55 mL) and acetic acid (77 µL) in a MeCN/MeOH (1:1 v/v, 3.0 mL) was stirred at r.t., for 24 h. The resulting mixture was dried under vacuum to obtain a red solid. This residue was dissolved in MeOH and purified by size exclusion chromatography (Sephadex® LH-20), dried under vacuum and lyophilized from water obtaining a red solid (22.0 mg; Kaiser Test 60 μmol_{NH2} g^{-1}), which is stored in a desiccator until characterization or use.

3. Supplementary Figures and Tables

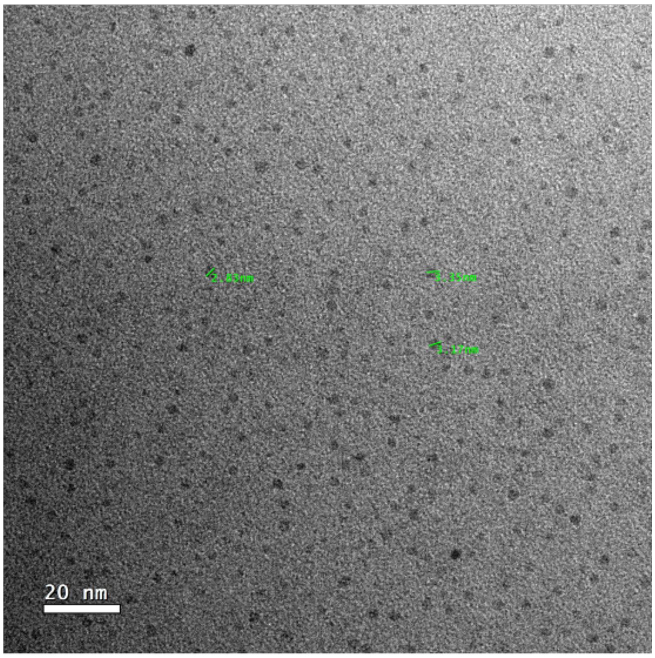

Figure S1. TEM of *p*B-NCDs. TEM micrograph from a drop-casted aqueous solution.

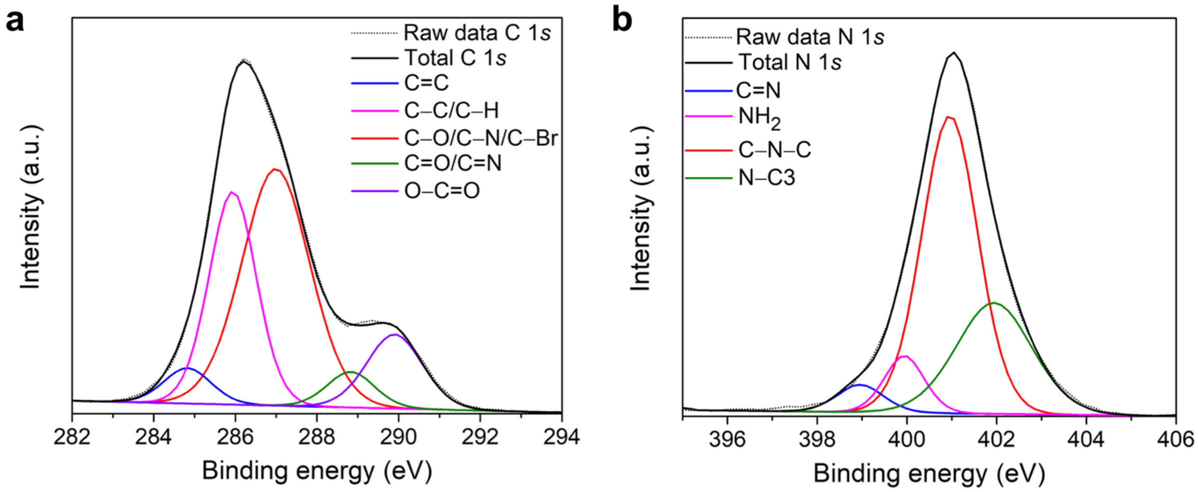

Figure S2. **XPS of *p*B-NCDs.** (**a**) deconvoluted C 1*s* spectra into five surface components corresponding to sp^2 (C=C) at 284.8 eV (5.0%), sp^3 (C–C, C–H) at 285.9 eV (29.8%), C–O/C–N at 286.9 eV (47.9%), C=O/C=N at 288.8 eV (5.0%), and a component at 289.9 eV (12.3%) attributed to O–C=O; (**b**) deconvoluted N 1*s* spectra into four peaks at 398.9 (4.5%), 399.9 (8.0%), 400.9 (58.2%), 401.9 (29.3%) eV corresponding to C=N, NH_2, C–N–C and N–C3, respectively.

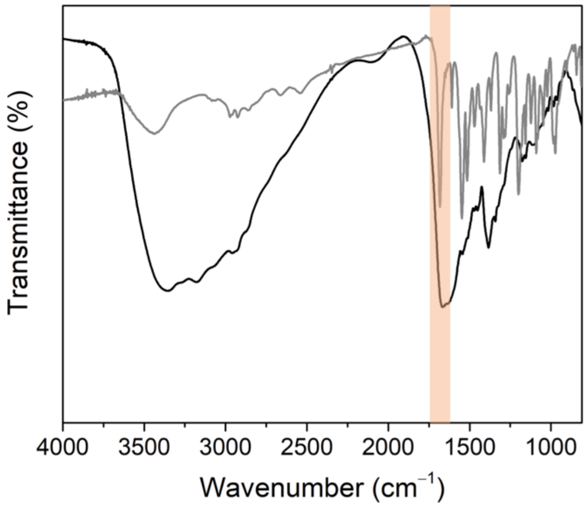

Figure S3. FT-IR spectra. *p*B-NCDs (black line) and BODIPY-*p*-COOH (grey line).

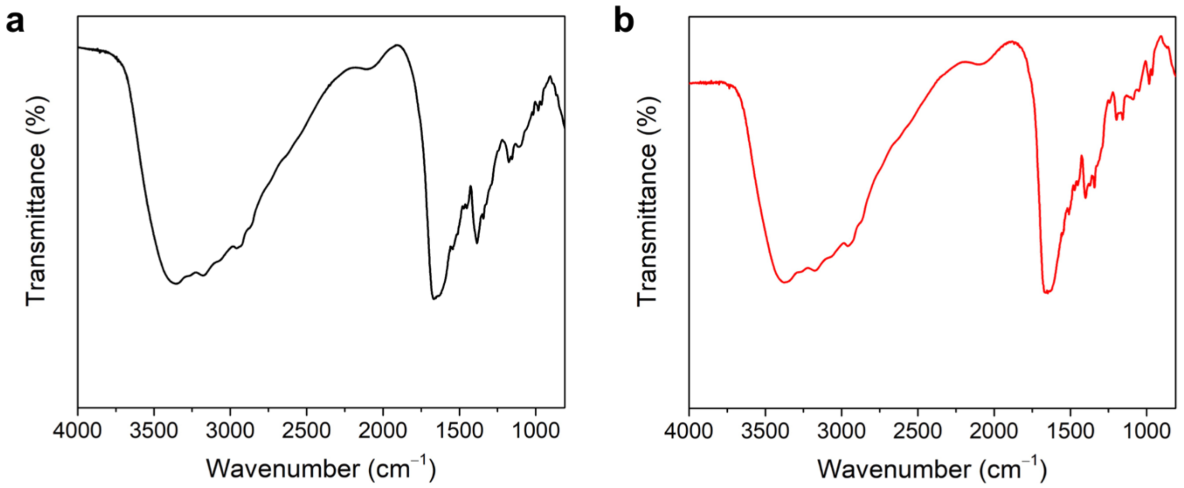

Figure S4. FT-IR spectra. (**a**) *p*B-NCDs and (**b**) *o*B-NCDs. Absorptions at 1669 and 1623 cm^{-1} show the presence of C=O bonds, while absorption at 1342 cm^{-1} confirms the presence of C–O. Additionally, peaks at 1178, 1155 and 1108 cm^{-1} display C–O–C bonds, and the broad peak at 3353 cm^{-1} reveal O–H/N–H bonding. Absorptions at 2945 and 1385 cm^{-1} are indicative of C–H and B–N stretching, respectively. Furthermore, C=N/C=C (1545 and 1511 cm^{-1}) and C–N (1470 and 1452 cm^{-1}) functional groups can be identified.

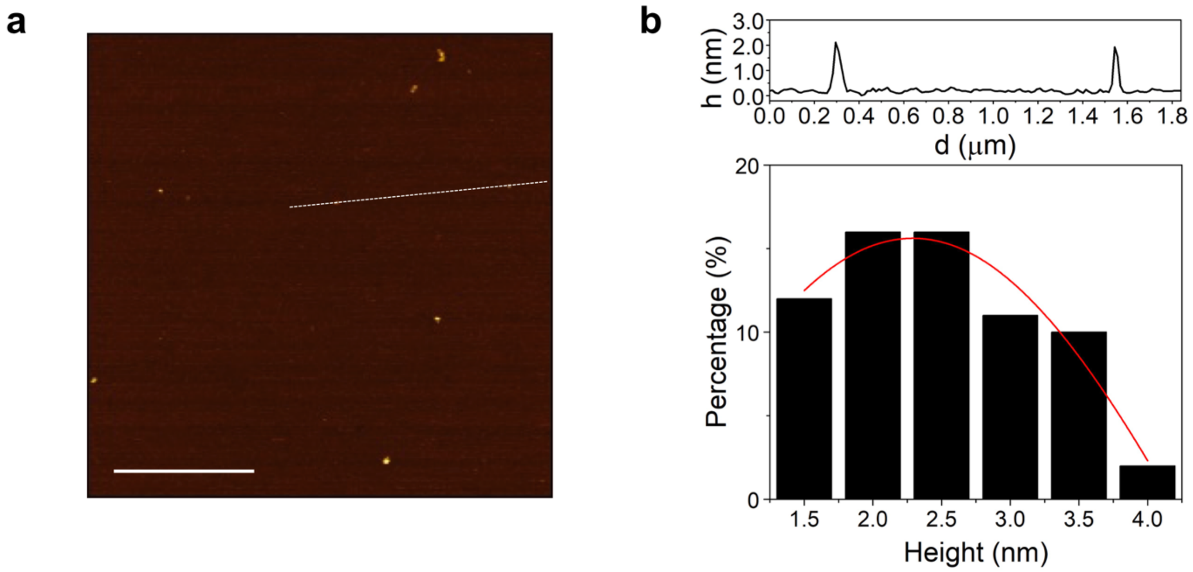

Figure S5. AFM of *o*B-NCDs. (**a**) Tapping mode AFM (3.3 × 3.3 μm) from a drop-casted aqueous solution on a mica substrate (scale bar, 1 μm); (**b**) height profile along the dashed line and size histogram with curve fit using a Gaussian model.

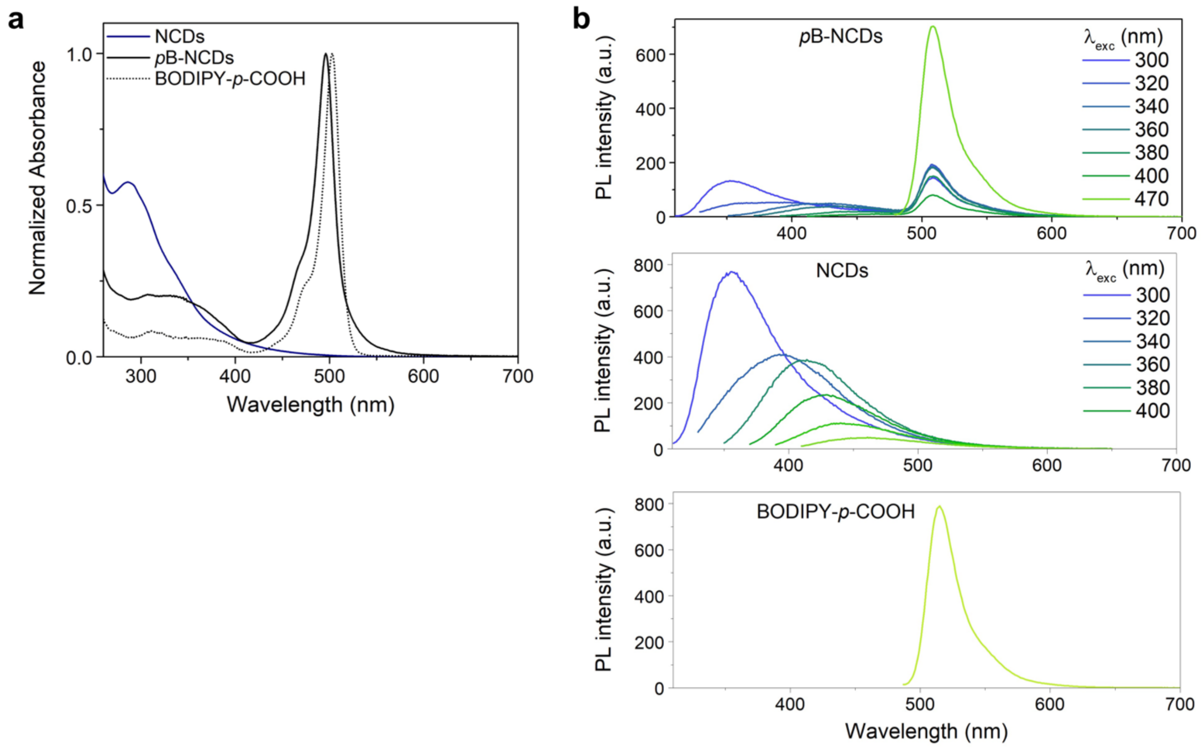

Figure S6. UV-Vis and PL emission spectra. (**a**) Normalized UV-Vis spectra of NCDs in water (blue line), *p*B-NCDs water (black line) and BODIPY-*p*-COOH in DMSO (dashed black line) at 298 K; (**b**) PL emission spectra at different excitation wavelengths of *p*B-NCDs in water (up), NCDs in water (middle) and BODIPY-*p*-COOH in DMSO (down).

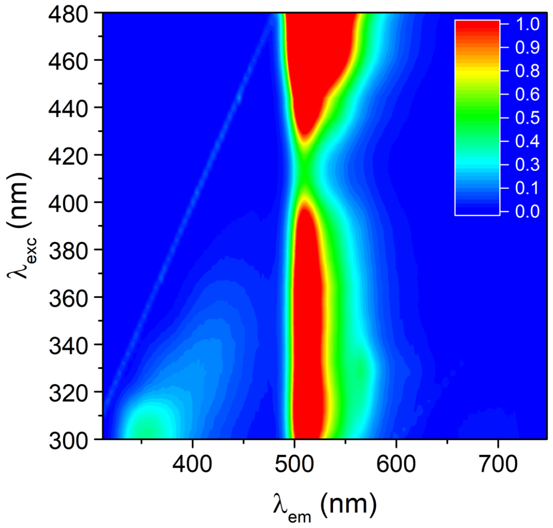

Figure S7. PL matrix of *p*B-NCDs showing the fluorescence mapping by exciting at different wavelengths.

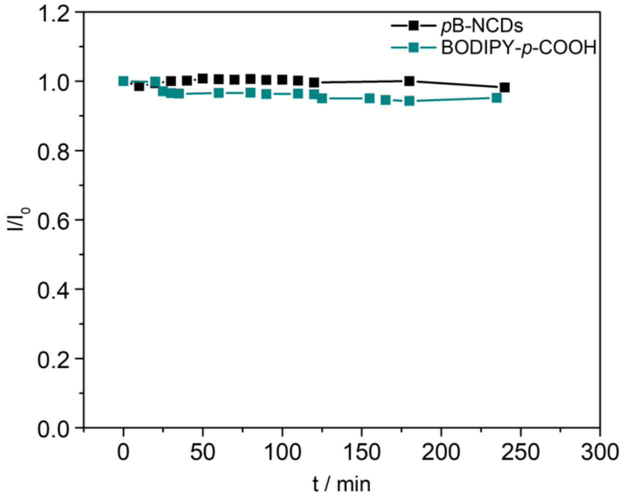

Figure S8. Normalized PL intensity variations of *p*B-NCDs and BODIPY-*p*-COOH. Normalized PL peak intensity at 507 nm over 4 hours of irradiation of *p*B-NCDs in water and BODIPY-*p*-COOH in DMSO (using the excitation light of the spectrometer).

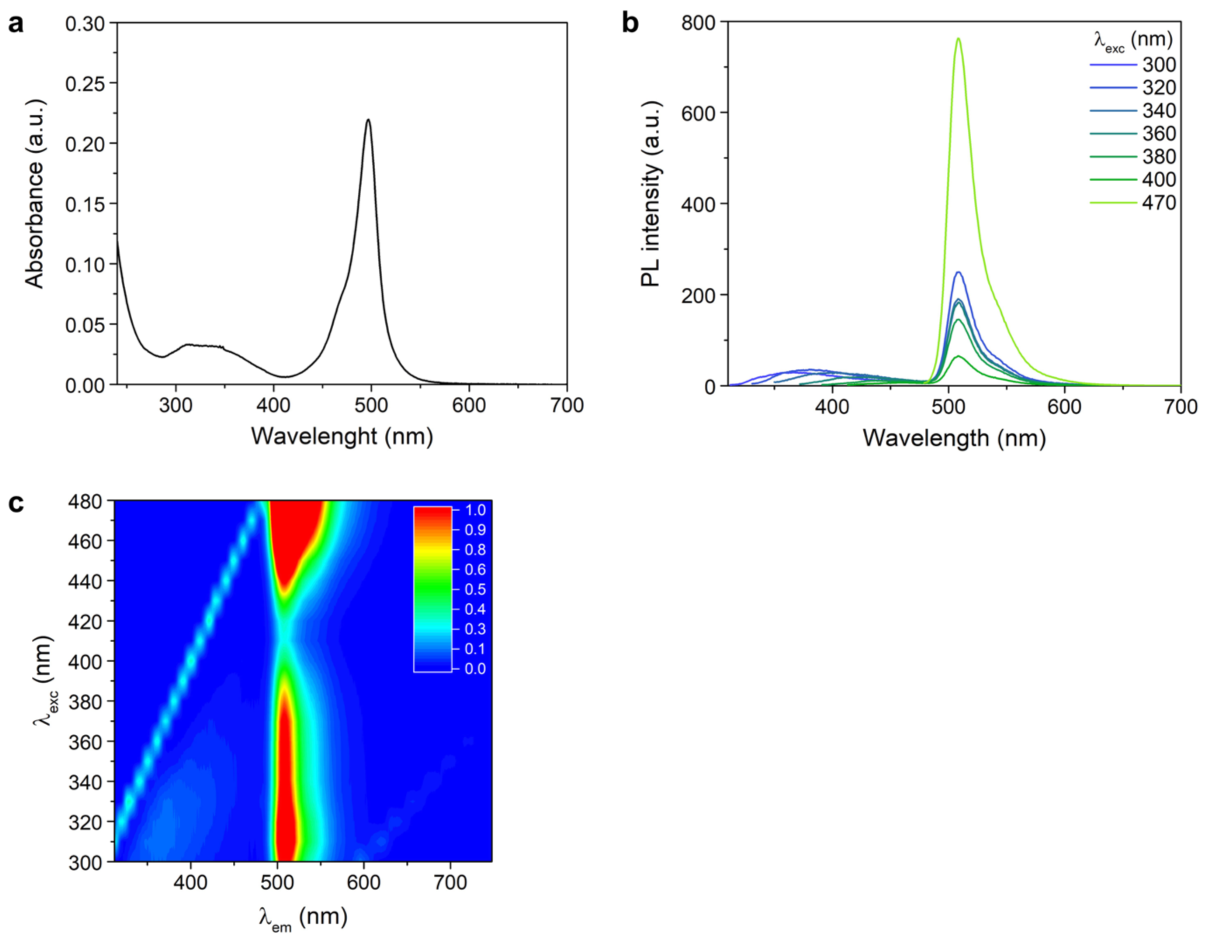

Figure S9. Photophysical characterization of *o*B-NCDs. Experiments performed in water at 298 K. (**a**) UV-Vis spectrum (2.8×10^{-2} mg mL^{-1}); (**b**) PL emission spectra at different excitation wavelengths; (**c**) PL matrix showing the fluorescence mapping by exciting at different wavelengths.

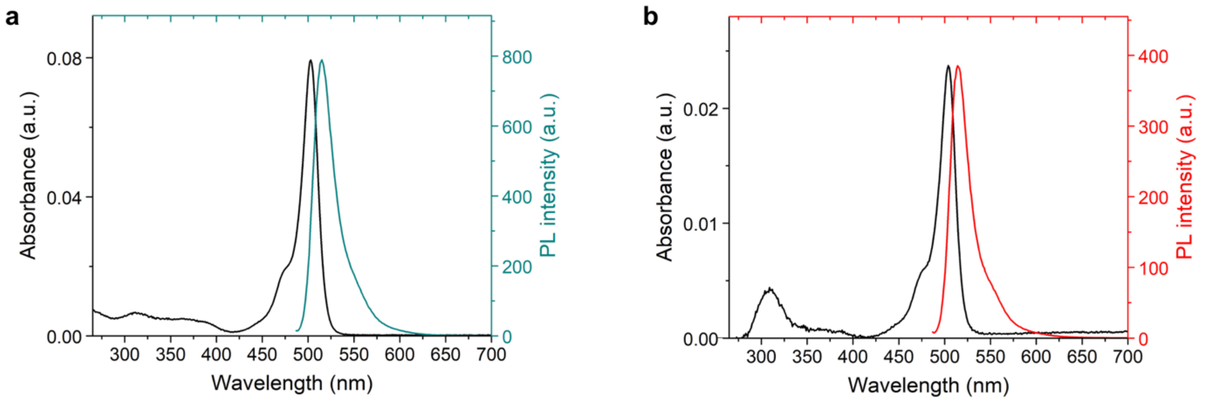

Figure S10. UV-Vis and PL emission spectra in DMSO at 298 K of (**a**) BODIPY-*p*-COOH (1.21 μM); (**b**) BODIPY-*o*-COOH (0.54 μM).

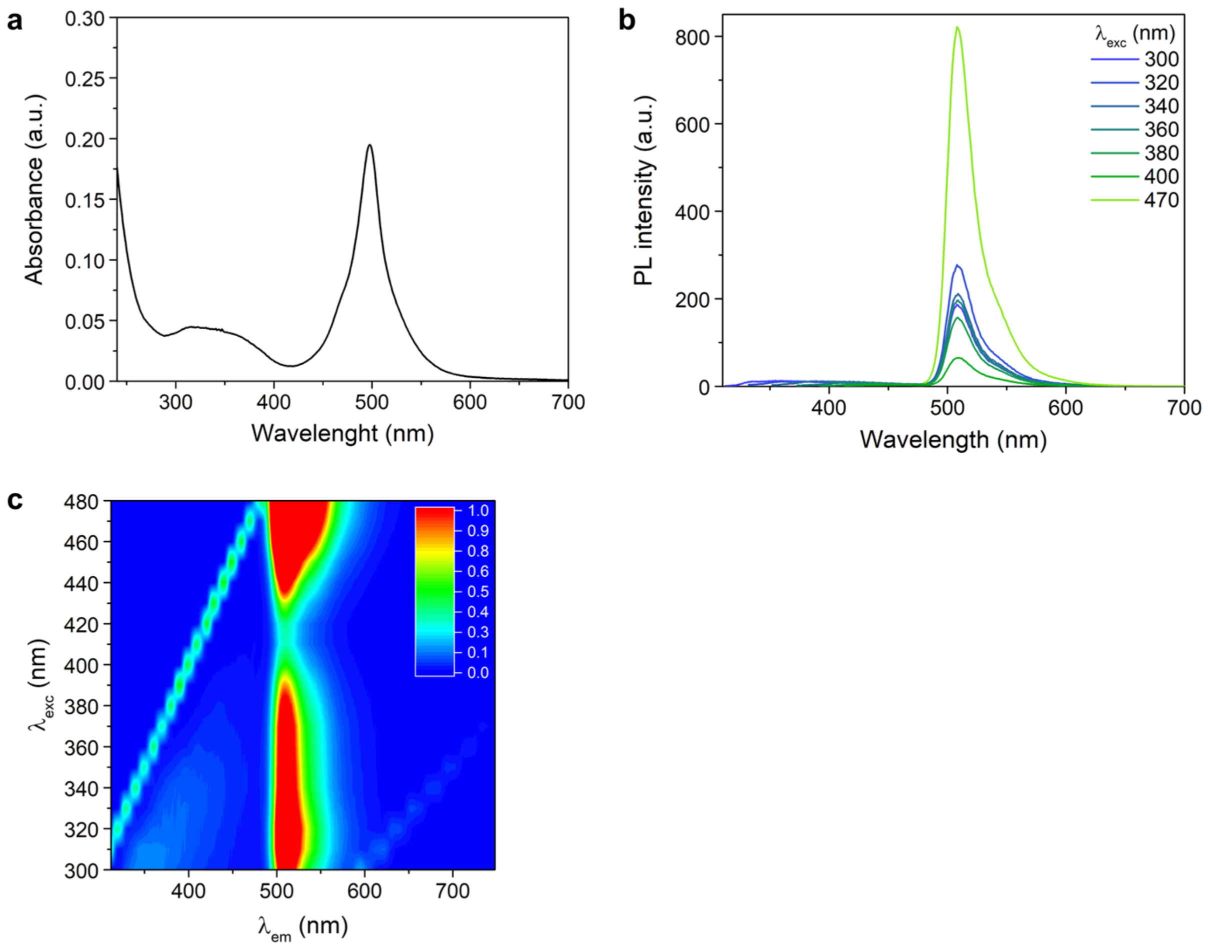

Figure S11. Photophysical characterization of methylated *o*B-NCDs. Experiments performed in water at 298 K. (**a**) UV-Vis spectrum (8.2×10^{-2} mg mL^{-1}); (**b**) PL emission spectra at different excitation wavelengths; (**c**) PL matrix showing the fluorescence mapping by exciting at different wavelengths.

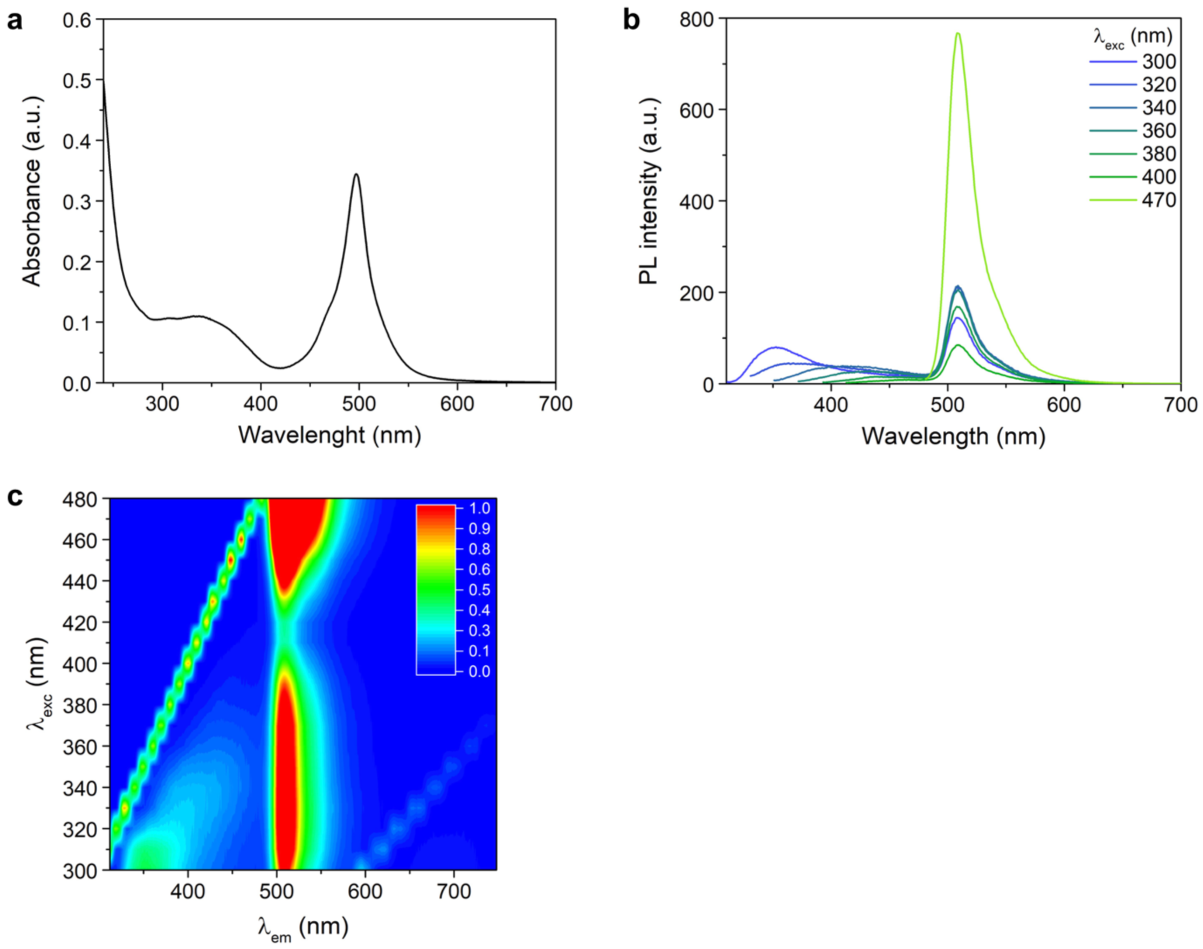

Figure S12. Photophysical characterization of methylated *p*B-NCDs. Experiments performed in water at 298 K. (**a**) UV-Vis spectrum (0.56 mg mL^{-1}); (**b**) PL emission spectra at different excitation wavelengths; (**c**) PL matrix showing the fluorescence mapping by exciting at different wavelengths.

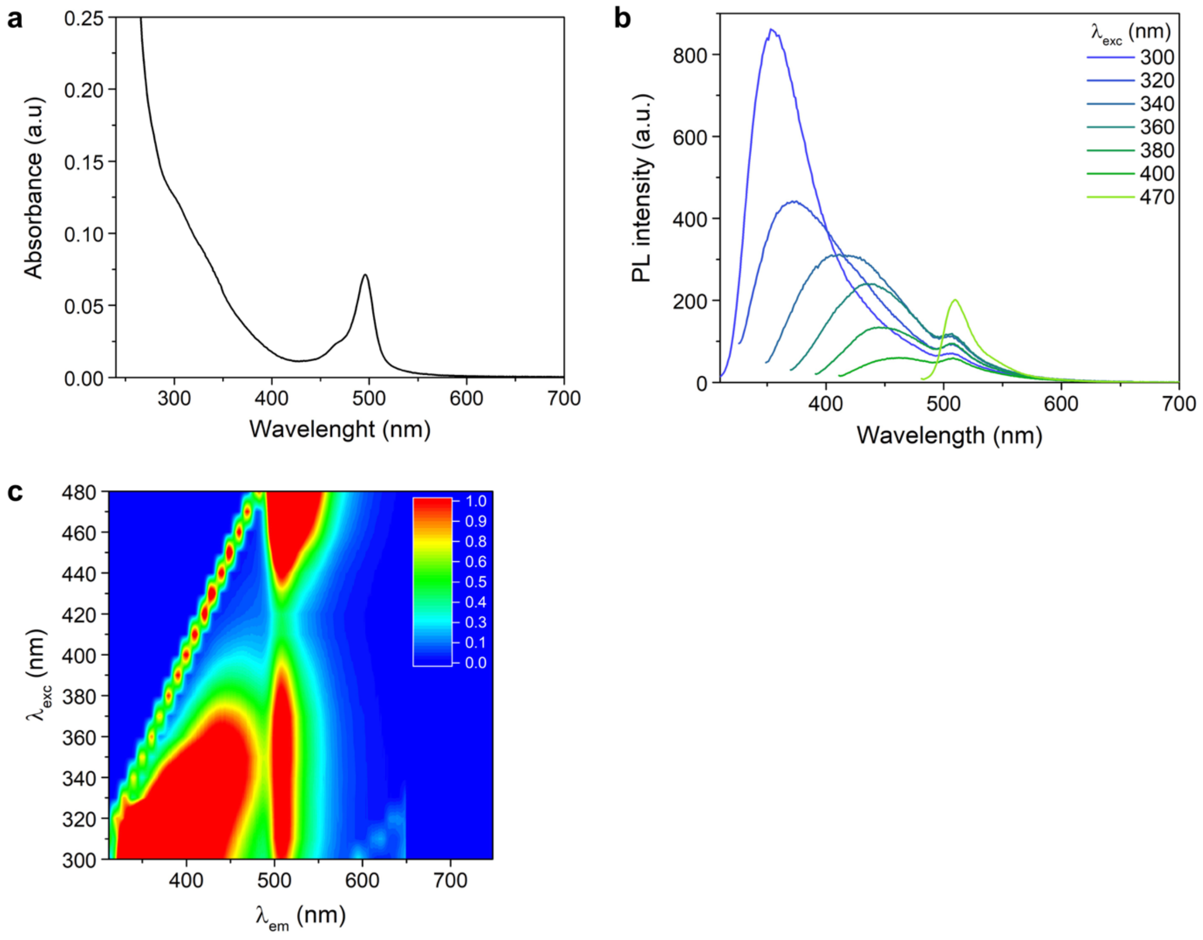

Figure S13. Photophysical characterization of propylated *p*B-NCDs. Experiments performed in water at 298 K. (**a**) UV-Vis spectrum (1.18 mg mL^{-1}); (**b**) PL emission spectra at different excitation wavelengths; (**c**) PL matrix showing the fluorescence mapping by exciting at different wavelengths.

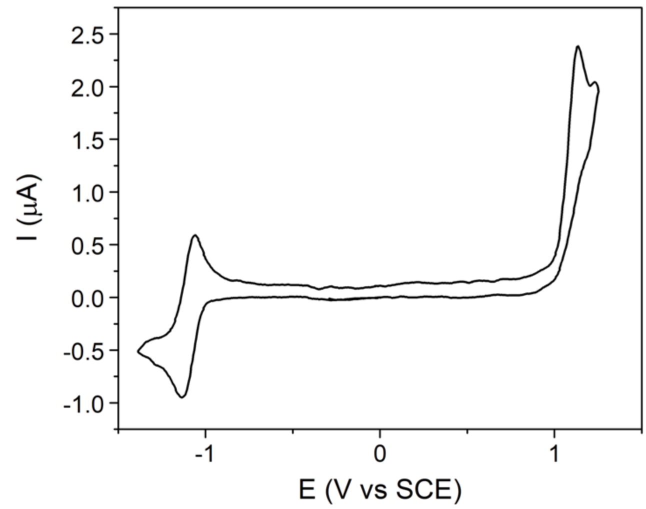

Figure S14. Cyclic Voltammetry of BODIPY-*p*-COOH (1.0 mM) in DMF/0.1 M Bu_4NPF_6, scanning the potential between +1.25 V and −1.4 V at scan rate 0.05 V s^{-1}.

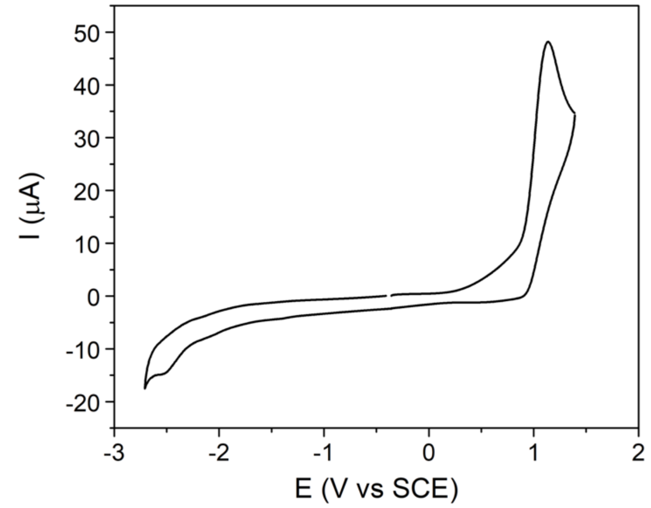

Figure S15. Cyclic Voltammetry of NCDs (1.0 mg mL^{-1}), in DMF/0.1 M Bu_4NPF_6, scanning the potential between +1.4 V and −2.7 V at scan rate 0.1 V s^{-1}.

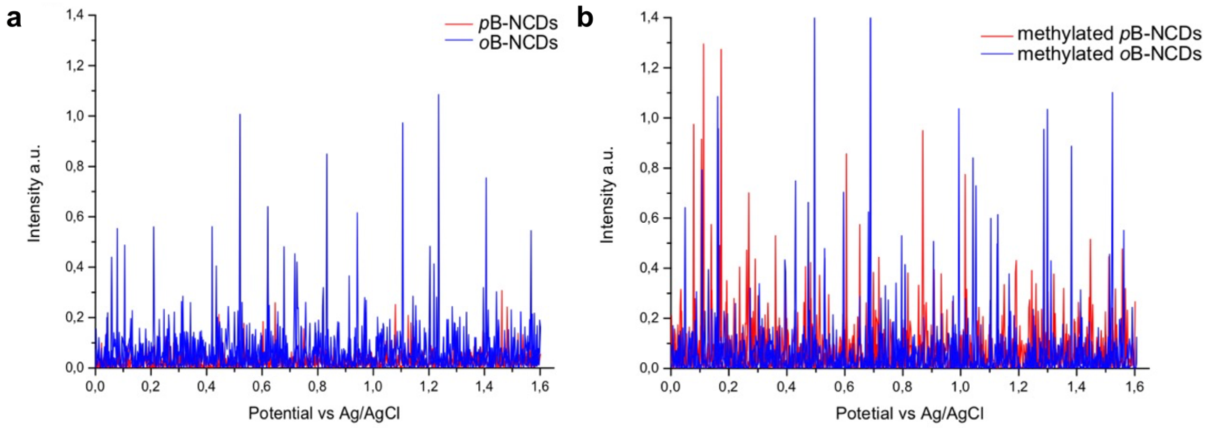

Figure S16. ECL intensities vs potential of (**a**) *p*B-NCDs (1.0 mg mL^{-1}, red line) and *o*B-NCDs (1.0 mg mL^{-1}, blue line) and (**b**) methylated *p*B-NCDs (1.0 mg mL^{-1}, red line) and methylated *o*B-NCDs (1.0 mg mL^{-1}, blue line). Experiments performed in phosphate buffer 0.2 м, pH = 6.9. Potential applied of 0 to 1.6 V, scan rate 0.1 V s^{-1}. Glassy Carbon electrode referred to Ag/AgCl. Pt spiral as counter electrode. Photomultiplier bias 750 V.

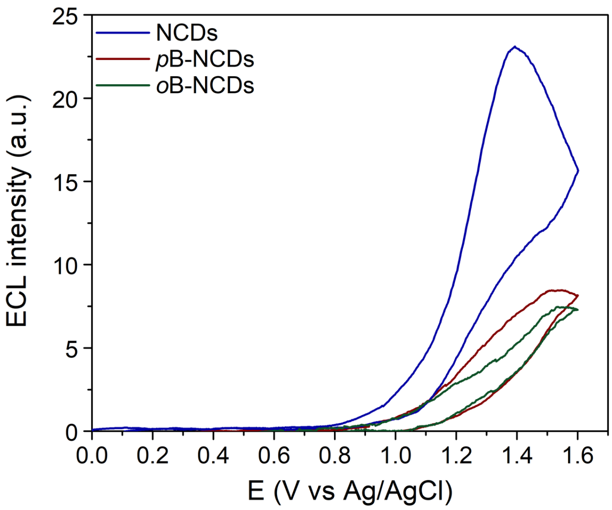

Figure S17. ECL potential curves of *p*B-NCDs (1.0 mg mL^{-1}, wine line), NCDs (1.0 mg mL^{-1}, blue line) and *o*B-NCDs (1.0 mg mL^{-1}, green line). Experiments performed with TPrA 180 mM as coreactant in phosphate buffer 0.2 M as supporting electrolyte. Cyclic voltammetric analysis scanning from 0 to 1.6 V, scan rate 0.1 V s^{-1}. GC electrode potential referred to Ag/AgCl at room temperature. Platinum wire as counter electrode.

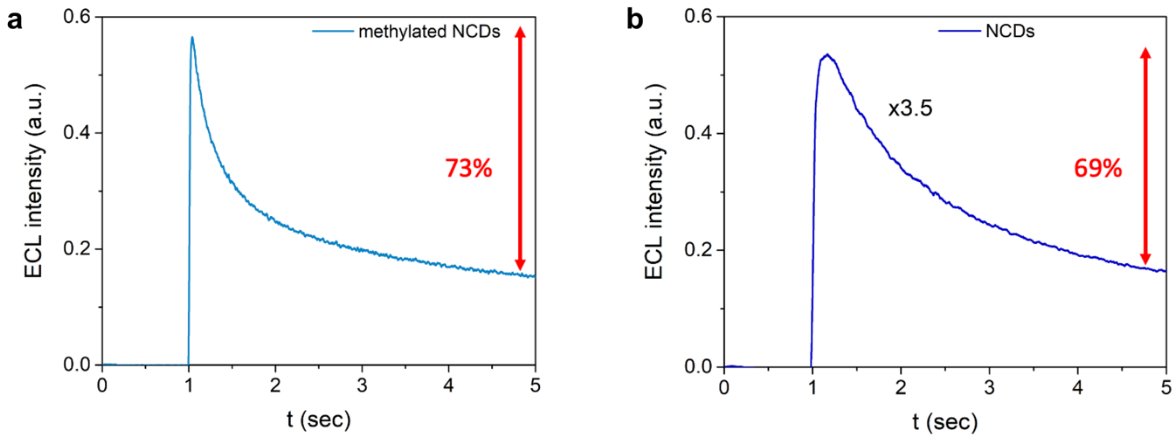

Figure S18. ECL vs time curves of (**a**) methylated NCDs (1.0 mg mL^{-1}, blue line) and (**b**) NCDs (1.0 mg mL^{-1}, dark blue line). Notice that the ECL intensity of NCDs is multiplied for a factor 3.5 for easy comparison with the ECL intensity of methylated NCDs. ECL intensities of methylated NCDs and NCDs show a drop of 73% and 69%, respectively. Experiments performed with TPrA 180 mM as coreactant in phosphate buffer 0.2 M as supporting electrolyte. Potential applied of 1.6 V. GC electrode potential referred to Ag/AgCl at room temperature. Platinum wire as counter electrode.

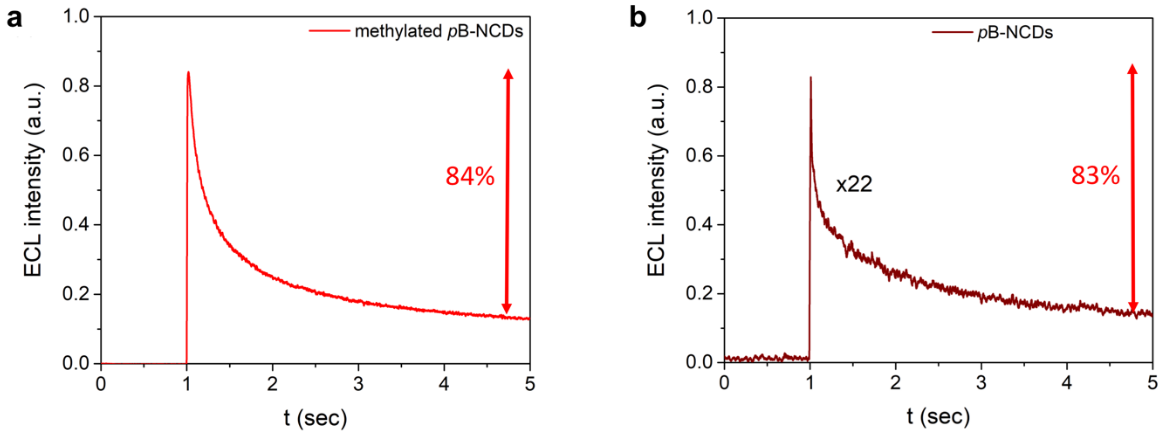

Figure S19. ECL vs time curves of (**a**) methylated *p*B-NCDs (1.0 mg mL^{-1}, red line) and (**b**) *p*B-NCDs (1.0 mg mL^{-1}, wine line). Notice that the ECL intensity of *p*B-NCDs is multiplied for a factor 22 for easy comparison with the ECL intensity of methylated *p*B-NCDs. ECL intensities of methylated *p*B-NCDs and *p*B-NCDs show a drop of 84% and 83%, respectively. Experiments performed with TPrA 180 mM as coreactant in phosphate buffer 0.2 M as supporting electrolyte. Potential applied of 1.6 V. GC electrode potential referred to Ag/AgCl at room temperature. Platinum wire as counter electrode.

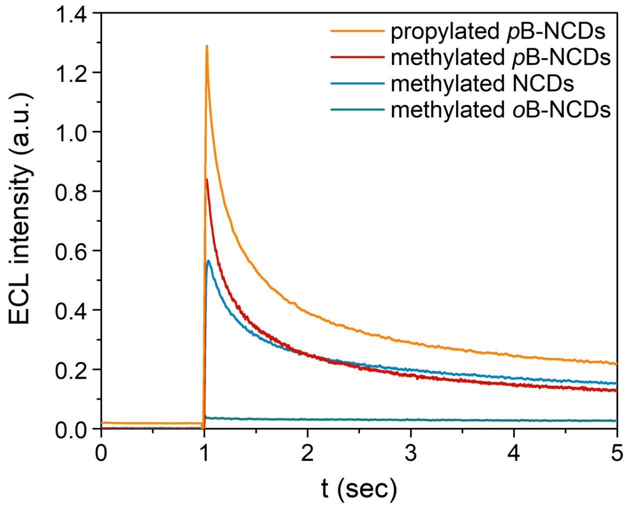

Figure S20. ECL vs time curves of methylated *p*B-NCDs (1.0 mg mL^{-1}, red line), methylated NCDs (1.0 mg mL^{-1}, blue line), methylated *o*B-NCDs (1.0 mg mL^{-1}, green line) and propylated *p*B-NCDs (1.0 mg mL^{-1}, orange line). Experiments performed with TPrA 180 mM as coreactant in phosphate buffer 0.2 M as supporting electrolyte. Potential applied of 1.6 V. GC electrode potential referred to Ag/AgCl at room temperature. Platinum wire as counter electrode.

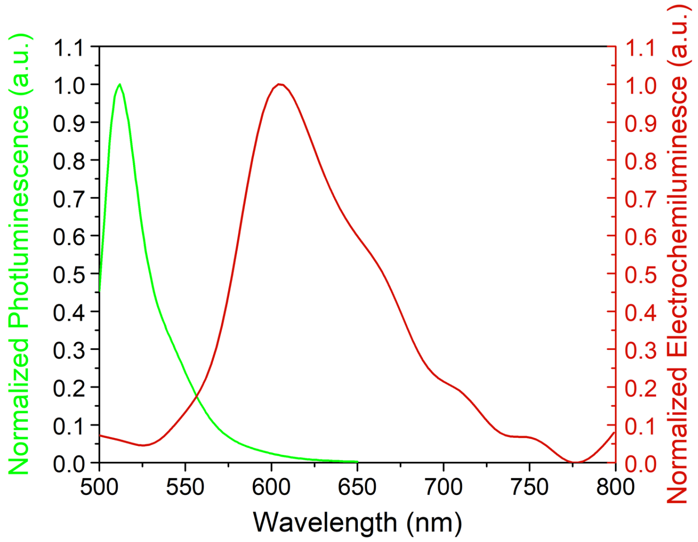

Figure S21. Maximum emission wavelength of normalized photoluminescence relative to methylated *p*B-NCDs (0.5 mg mL^{-1} in water, green line) compared to maximum wavelength of normalized electrochemiluminescence relative to the same sample methylated *p*B-NCDs (1.0 mg mL^{-1} with TPrA 180 mM in PBS 0.2 M, red line).

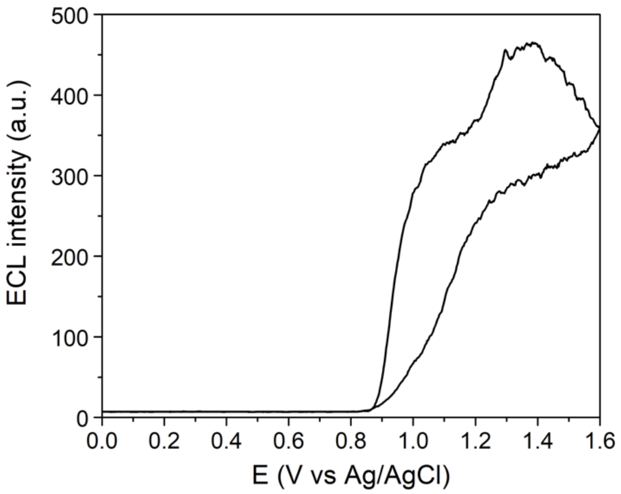

Figure S22. ECL potential curve of $[Ru(bpy)_3]^{2+}$. Experiments performed with TPrA 180 mM as coreactant in phosphate buffer 0.2 M as supporting electrolyte. Cyclic voltammetric analysis scanning from 0 V to 1.6 V, scan rate 0.1 V s^{-1}. GC electrode potential referred to Ag/AgCl at room temperature. Platinum wire as counter electrode.

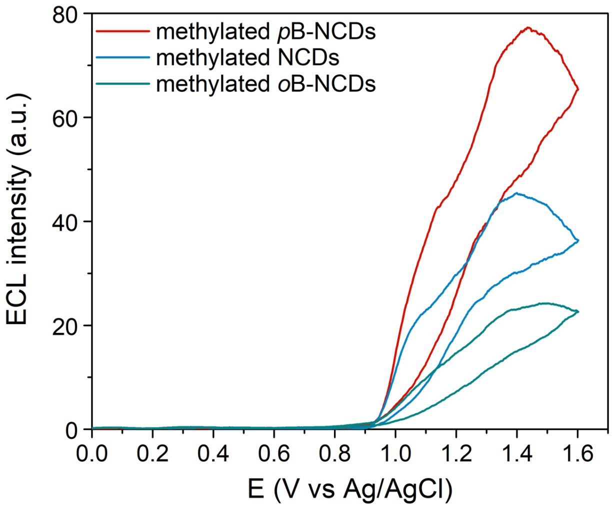

Figure S23. ECL potential curves of methylated *p*B-NCDs (1.0 mg mL^{-1}, red line), methylated NCDs (1.0 mg mL^{-1}, blue line) and methylated *o*B-NCDs (1.0 mg mL^{-1}, green line). Experiments performed with TPrA 180 mM as coreactant in phosphate buffer 0.2 M as supporting electrolyte. Cyclic voltammetric analysis scanning from 0 V to 1.6 V, scan rate 0.1 V s^{-1}. GC electrode potential referred to Ag/AgCl at room temperature. Platinum wire as counter electrode.

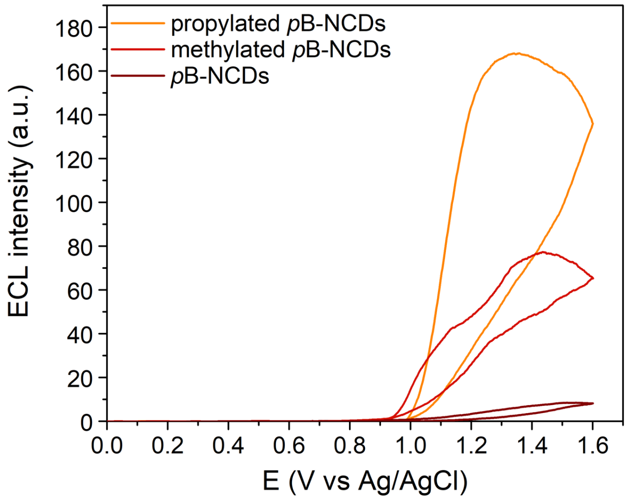

Figure S24. **ECL potential curves** of propylated *p*B-NCDs (1.0 mg mL^{-1}, orange line), methylated *p*B-NCDs (1.0 mg mL^{-1}, red line) and *p*B-NCDs (1.0 mg mL^{-1}, wine line). Experiments performed with TPrA 180 mM as coreactant in phosphate buffer 0.2 M as supporting electrolyte. Cyclic voltammetric analysis scanning from 0 V to 1.6 V, scan rate 0.1 V s^{-1}. GC electrode potential referred to Ag/AgCl at room temperature. Platinum wire as counter electrode.

Table S1. Comparison between the relative ECL efficiency for oxidative-reduction mechanism (Φ_{ECL}, see materials and methods for the details) performances of B-NCDs with other recently reported and highly active ECL dyes.

ECL emitter	Coreactant	Solvent	Φ_{ECL} (%)^b	Reference
$[Ru(bpy)_3]^{2+}$	TPrA	PB^a	100	-
$[(bpy)_2Ru](bphb)]^{2+}$	TPrA	PB^a	5.8	Richter et al., *Anal. Chem.* 1998
Copper metal cluster	TPrA	CH_2Cl_2	15	Han et al., *J. Electroanal. Chem.*, 2017
$[((phen)_2Ru(dpp))_2RhCl_2]^{5+}$	TPrA	Tris buffer	1.4	Wang et al., *Anal. Chem.* 2009
Spirofluorene Dye	TPrA	CH_2Cl_2	454	Li et al., *Chem. Eur. J.*, 2016
Boron-dipyrromethene (BODIPY)	TPrA	CH_3CN	44	P. Chauhan et al. *J. Electroanal. Chem.* 2016
Methylated *p*B-NCDs	TPrA	PB^a	13	This work
Propylated *p*B-NCDs	TPrA	PB^a	44	This work

a) PB is phosphate buffer; b) the ECL efficiency was calculated by comparison with $[Ru(bpy)_3]^{2+}$as reference (Φ_{ECL} = 100), see Figure S22.

Table S2. Comparison between the ECL efficiency performances, obtained in annihilation (Φ_{ECL}), of the BODIPY dye with other recently reported.

ECL emitter	Φ_{ECL} (%)^a	Reference
$[Ru(bpy)_3]^{2+}$	5	Rubinstein, L. et al., J. *Am. Chem. Soc.* 1981
Boron-dipyrromethene (BODIPY)	16	P. Chauhan et al., *J. of Electroanal. Chem.*, 2016
BOPEG2	20	A. B. Nepomnyashchii et al. *J. Phys. Chem. C*, 2013)
BODIPY1	21	A. B. Nepomnyashchii et al. *Acc. Chem. Res.*, 2012
BODIPY2	0.6	A. B. Nepomnyashchii et al. *Acc. Chem. Res*, 2012
BODIPY3	0.8	A. B. Nepomnyashchii et al. *Acc. Chem. Res*, 2012
BODIPY4	0.5	A. B. Nepomnyashchii et al. *Acc. Chem. Res*, 2012
BODIPY5	15	A. B. Nepomnyashchii et al. *Acc. Chem. Res*, 2012

a) the ECL efficiency in annihilation was calculated by comparison with $[Ru(bpy)_3]^{2+}$as reference (Φ_{ECL} = 5%).

Table S3: PL decay parameters (time constants τ_i and populations A_i) for NCDs, B-NCDs and BODIPY-COOH

**Sample**	**τ_1, ns (A_1)^a**	**τ_2, ns (A_2)^a**	**τ_3, ns (A_3)^a**	**$\langle\tau\rangle$, ns^a**	**τ_1, ns (A_1)^b**	**τ_2, ns (A_2)^b**	**τ_3, ns (A_3)^b**	**$\langle\tau\rangle$, ns^b**
NCDs	0.9 (12%)	4.5 (45%)	12.8 (43%)	7.6	-	-	-	-
Methylated NCDs	1.0 (13%)	5.5 (43%)	17.8 (44%)	10.3	-	-	-	-
BODIPY-*p*-COOH	3.4	-	-	-	3.7			
*p*B-NCDs	1.0 (9%)	3.5 (77%)	9.9 (14%)	4.2	0.3 (3%)	2.8 (73%)	5.1 (23%)	3.3
Methylated *p*B-NCDs	0.7 (4%)	3.3 (88%)	8.9 (8%)	3.6	0.2 (3%)	2.9 (73%)	4.6 (24%)	3.3
Propylated *p*B-NCDs	0.8 (17%)	3.7 (57%)	11.0 (26%)	5.1	0.2 (7%)	2.2 (63%)	6.6 (30%)	3.4

$A_1 + A_2 + A_3 = 1$. a) excitation wavelength at 340 nm; b) excitation wavelength at 445 nm.

4. References

[1] Cold Spring Harbor Laboratory, *Cold Spring Harb. Protoc.* **2008**, *2008*, https://doi.org/10.1101/pdb.rec11372.

[2] C. Würth, M. Grabolle, J. Pauli, M. Spieles, U. Resch-Genger, *Nat. Protoc.* **2013**, *8*, 1535.

[3] W. L. Wallace, A. J. Bard, *J. Phys. Chem.* **1979**, *83*, 1350.

[4] A. G. Riches, T. Cablewski, V. Glattauer, H. Thissen, L. Meagher, *Tetrahedron* **2012**, *68*, 9448.

[5] S. Iwaki, K. Hokamura, M. Ogawa, Y. Takehara, Y. Muramatsu, T. Yamane, K. Hirabayashi, Y. Morimoto, K. Hagisawa, K. Nakahara, T. Mineno, T. Terai, T. Komatsu, T. Ueno, K. Tamura, Y. Adachi, Y. Hirata, M. Arita, H. Arai, K. Umemura, T. Nagano, K. Hanaoka, *Org. Biomol. Chem.* **2014**, *12*, 8611.

[6] A. B. Nepomnyashchii, A. J. Pistner, A. J. Bard, J. Rosenthal, *J. Phys. Chem. C* **2013**, *117*, 5599.

[7] D. Wang, J. Fan, X. Gao, B. Wang, S. Sun, X. Peng, *J. Org. Chem.* **2009**, *74*, 7675.
